# All‐Around Universal and Photoelastic Self‐Healing Elastomer with High Toughness and Resilience

**DOI:** 10.1002/advs.202103235

**Published:** 2021-10-18

**Authors:** Jarkko Tolvanen, Mikko Nelo, Jari Hannu, Jari Juuti, Heli Jantunen

**Affiliations:** ^1^ Microelectronics Research Unit Faculty of Information Technology and Electrical Engineering University of Oulu P.O. Box 4500 Oulu FIN‐90014 Finland

**Keywords:** autonomous healing, hydroxypropyl cellulose, liquid crystal, mechanochromic, strain sensors, supramolecular, underwater self‐healing

## Abstract

Ultimately soft electronics seek affordable and high mechanical performance universal self‐healing materials that can autonomously heal in harsh environments within short times scales. As of now, such features are not found in a single material. Herein, interpenetrated elastomer network with bimodal chain length distribution showing rapid autonomous healing in universal conditions (<7200 s) with high efficiency (up to 97.6 ± 4.8%) is reported. The bimodal elastomer displays strain‐induced photoelastic effect and reinforcement which is responsible for its remarkable mechanical robustness (≈5.5 MPa stress at break and toughness ≈30 MJ m^−3^). The entropy‐driven elasticity allows an unprecedented shape recovery efficiency (100%) even after fracturing and 100% resiliency up to its stretching limit (≈2000% strain). The elastomers can be mechanically conditioned leading to a state where they recover their shape extremely quickly after removal of stress (nearly order of magnitude faster than pristine elastomers). As a proof of concept, universal self‐healing mechanochromic strain sensor is developed capable of operating in various environmental conditions and of changing its photonic band gap under mechanical stress.

## Graphical abstract

A versatile, extremely tough, and resilient universal self‐healing elastomer is achieved with bimodal interpenetrated network design. Elastomer shows a high toughness up to 30 MJ m^−3^ while being 100% resilient up to its stretching limit (≈2000% strain). The material shows high self‐healing efficiencies (up to 97.6 ± 4.8%) even in harsh environmental conditions including supercooled saline water (−19 **°**C).

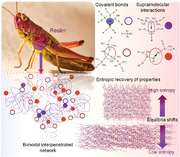

## 1 Introduction

Soft materials inspired by biological systems hold great promise for soft robotics, prostheses, and implantable and skin‐mountable electronics. Features such as compliance and conformability are important in order for robotic systems and prostheses to have the ability to perform difficult manipulation tasks in ever‐changing environments and for safe physical interaction with human beings.^[^ ^1^, ^2^, ^3^ ^]^ In implantable and skin‐interfaced electronics, soft materials may not only enable more unobtrusive sensor technologies, but may also have other advantages (improved signal‐to‐noise ratio, sensitivity, etc.) in comparison to their rigid counterparts.^[^ ^4^, ^5^, ^6^, ^7^, ^8^ ^]^ Although thin and compliant soft systems are deformable and resistant to blunt damage, they have poor mechanical damage resistance to punctures, tears, and scratches. Moreover, defects such as dislocations and crystalline boundaries in soft materials distribute mechanical stress nonuniformly. These give rise to macroscopic damages on fatigue that may propagate further, and eventually lead to mechanical failure. On the other hand, harsh and ever‐changing environmental conditions pose significant risks for the longevity of these devices. Thus, great demand exists for universal self‐healing materials meeting industrial standards of mechanical performance.

Since the appearance of self‐healing polymers, variety nonautonomous and autonomous design strategies have been developed.^[^ ^9^, ^10^, ^11^, ^12^, ^13^ ^]^ Self‐healing mechanisms relying on encapsulated or microvascular networks containing healing agents may find a specific limited use for mechanically robust soft systems but their use more generally poses apparent challenges.^[^ ^3^, ^14^, ^15^ ^]^ A more promising strategy has been to use polymeric systems healing on an energy input trigger or autonomously without intervention.^[^ ^16^, ^17^, ^18^, ^19^, ^20^, ^21^, ^22^, ^23^, ^24^ ^]^ Some of the most effective strategies have been based on supramolecular interactions, or more recently, even on shape memory effects.^[^ ^3^, ^15^, ^25^, ^26^ ^]^ However, universal self‐healing materials capable of operating under wide range of harsh environments are still at scarce.^[^ ^21^ ^]^

Ultimately, soft material systems are striving for versatile, or in other words, “all‐around” universal self‐healing materials. In soft materials, high mechanical performance is a combination of properties including high strength, toughness, and resilience, while transparency in visible light spectrum is a must for optoelectronic and smart skin applications. Achieving the above‐mentioned properties with a single material without compromising a fast autonomous self‐healing ability even in harsh environments is extremely challenging in the light of the current knowledge of the viable design strategies. Through recent breakthroughs in autonomous self‐healing materials, mechanical strengths of the materials have even exceeded 40 MPa.^[^ ^9^, ^11^, ^12^, ^27^ ^]^ However, materials still lack resilience (despite their mechanical robustness) which is a key feature for soft electronics, and their self‐healing fails in harsh environmental conditions (faced in marine environments, polar regions, and so forth) due to its poor resistance. Vice versa, even though “optimizing” self‐healing properties through combination chemistries (or by other strategies) may enable materials to function in these challenging conditions, improvement comes at the expense of the overall mechanical performance.^[^ ^17^, ^21^, ^28^, ^29^ ^]^ Also, a major restriction in most self‐healing materials (for commercial applications of these materials) is their long healing times and partial recovery. This compromises the mechanical integrity as materials are very vulnerably during their slow restorative process, whereas without high self‐healing efficiency mechanical properties gradually weaken over‐time. Thus, new design strategies should be sought to develop materials with combination of high mechanical performance and autonomous universal self‐healing functionality.

**Figure 1:**
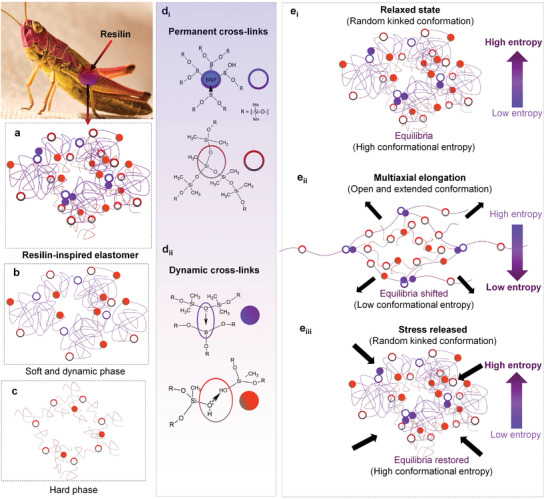
Schematic of universal self‐healing elastomer inspired by composites found in locusts. a–c) Schematic illustration of the bimodal interpenetrated network, where (a) corresponds to the cross‐linked poly(boronsiloxane)‐dimethylvinyl‐terminated dimethylsiloxane‐based elastomer, b) dynamic phase with long polymer chains, and c) a hard phase with short polymer chains. The similar backbone structures allow formation of interpenetrated 3D network with permanent net‐/junction points. d) The chemical structure is composed of d_i_) covalent bonds and d_ii_) dipole–dipole interactions. The polymer chains, covalent bonds (Si—O—Si and Si—O—B—O—Si), dipole–dipole interactions (O—B and O—H bonds) are presented by lines, circles, and spheres, respectively. e_i–iii_) Schematic illustration of the deformation mechanism with external force. e_i_) In relaxed state, the system has high conformational entropy. e_ii_) External force leads to shifting of equilibria and obtaining state of low conformational entropy. e_iii_) The conformational entropy switches from low to high state on removal of external force as elastomer retracts to its original shape.

Nature is a great source of inspiration for synthetic materials with high resilience. The elastomeric protein known as resilin has very remarkable elasticity as it enables powerful leg propulsion in insects.^[^ ^30^, ^31^, ^32^, ^33^ ^]^ Following the design concept of composites formed by resilin and chitinous cuticle in the locust, the combination of soft and hard phase has been mimicked in a universal self‐healing elastomer (**Figure** **1**). It was assumed that a soft and dynamic phase could act as an entropy spring with the existence of flexible and long chains connected by permanent junction points allowing large and reversible deformations to take place.

## 2 Results and Discussion

### 2.1 Resilin‐Inspired Bimodal Self‐Healing Elastomer

Synthesis of the bimodal interpenetrated elastomer network is illustrated in Figure 1a–d (description in the Experimental Section). Similar polymer backbone structures (Figures S1 and S2, Supporting Information) with cross‐linking elements allow the formation of permanent net‐/junction‐points (Figure 1b,c). These are formed by covalent bonds and interpenetrating networks, both of which have a role in the entropy‐driven elasticity by preventing the occurrence of chain slippage and flow (Figure 1d_i_). The dynamic chemical cross‐links consist of numerous dipole–dipole interactions (Figure 1d_ii_) allowing rapid and efficient universal self‐healing even in harsh environmental conditions (as later seen). During cross‐linking, the elastomer takes its permanent shape which then becomes its equilibrium.

The analogy of the bimodal interpenetrated elastomer network is similar to the isotropic elastomeric proteins and resilin‐chitinous cuticle composites found in insects. The function of the soft phase is to protect a hard phase from fracturing, while the hard phase provides structural integrity and allows entropy‐driven elasticity governed by the conformational entropy (associated with a particular structure) (Section S1, Supporting Information). In addition, the bimodal chain length distribution and chemisorption of nanoparticles were found to reinforce and improve the mechanical performance (as discussed later on). In the relaxed state, the system is in equilibrium because the long polymer chains (of the soft phase) with freely rotating links are in their energetically favorable conformation.^[^ ^34^ ^]^ The conformation has a high degree of freedom as it can be distributed in various randomly kinked and oriented conformations (Figure 1e_i_). Displacement of net‐/junction‐points exists under application of a force and an energetically unfavorable conformation (compared to the relaxed state) is occupied as equilibrium shifts. Deformed bimodal polymer chains have low probability to occupy large number of conformations (with both open and extended conformations) thus reducing conformational entropy (Figure 1e_ii_). The system is driven by the gain of conformational entropy when the force is removed to recover the energetically favorable conformation and restore the equilibrium.

### 2.2 Material Characterization

The elastomers were successfully synthetized as indicated by Fourier‐transform infrared spectroscopy (FTIR; Figure S2, Supporting Information). For instance, the FTIR spectra of a pristine elastomer in a static state featured negligible Si‐O:B dative bonded absorption peak intensities at 1340 cm^−1^ and two O—H peaks were absent at 3700 and 3290 cm^−1^ that correspond to nonhydrogen and intermolecular hydrogen bonded bands, respectively (indication that the condensation reaction was complete).^[^ ^35^, ^36^, ^37^ ^]^ Also, the characteristic peaks intensities of siloxane backbone were featured for Si(CH_3_)_2_‐ at 1260 cm^−1^ and Si(CH_3_)_2_‐O‐Si(CH_3_)_2_‐ at 1020–1090 cm^−1^.^[^ ^36^ ^]^ In dynamic state, the FTIR spectra of the elastomer show significant peak intensity changes (in comparison to static state) at 865 and 700 cm^−1^ that correspond to increased supramolecular interactions by Si‐O:B dative bonding. The peak intensity change at 1340 cm^−1^ indicates that boron‐containing functional groups are reforming bonds with siloxane backbone (in addition to peak intensity change at 3290 cm^−1^).^[^ ^35^, ^36^, ^37^ ^]^

The elastomers displayed an excellent thermal stability in thermogravimetric analysis (TGA) as decomposition started at temperatures exceeding 400 °C (Figure S3a, Supporting Information). The elastomers showed insignificant weight loss (less than 1%) up to 300 °C even though elastomers were in their hydrated state due to ambient humidity (a phenomenon discussed more later on). During heating process, endothermic peaks were observed in differential scanning calorimetry (DSC) close to decomposition temperatures and no exothermic peaks were found due to obvious reasons (i.e., temperature range of the DSC was limited to temperatures exceeding a room temperature) (Figure S3b, Supporting Information). However, we hypothesize that further studies may find evidence of the expected phase separation of soft and hard phase by observing two melting transitions (as seen in thermoplastic polycaprolactone–polyurethanes fibers)^[^ ^38^ ^]^ near melting transition of poly(dimethylsiloxane).

### 2.3 Mechanical Characterization

**Figure 2:**
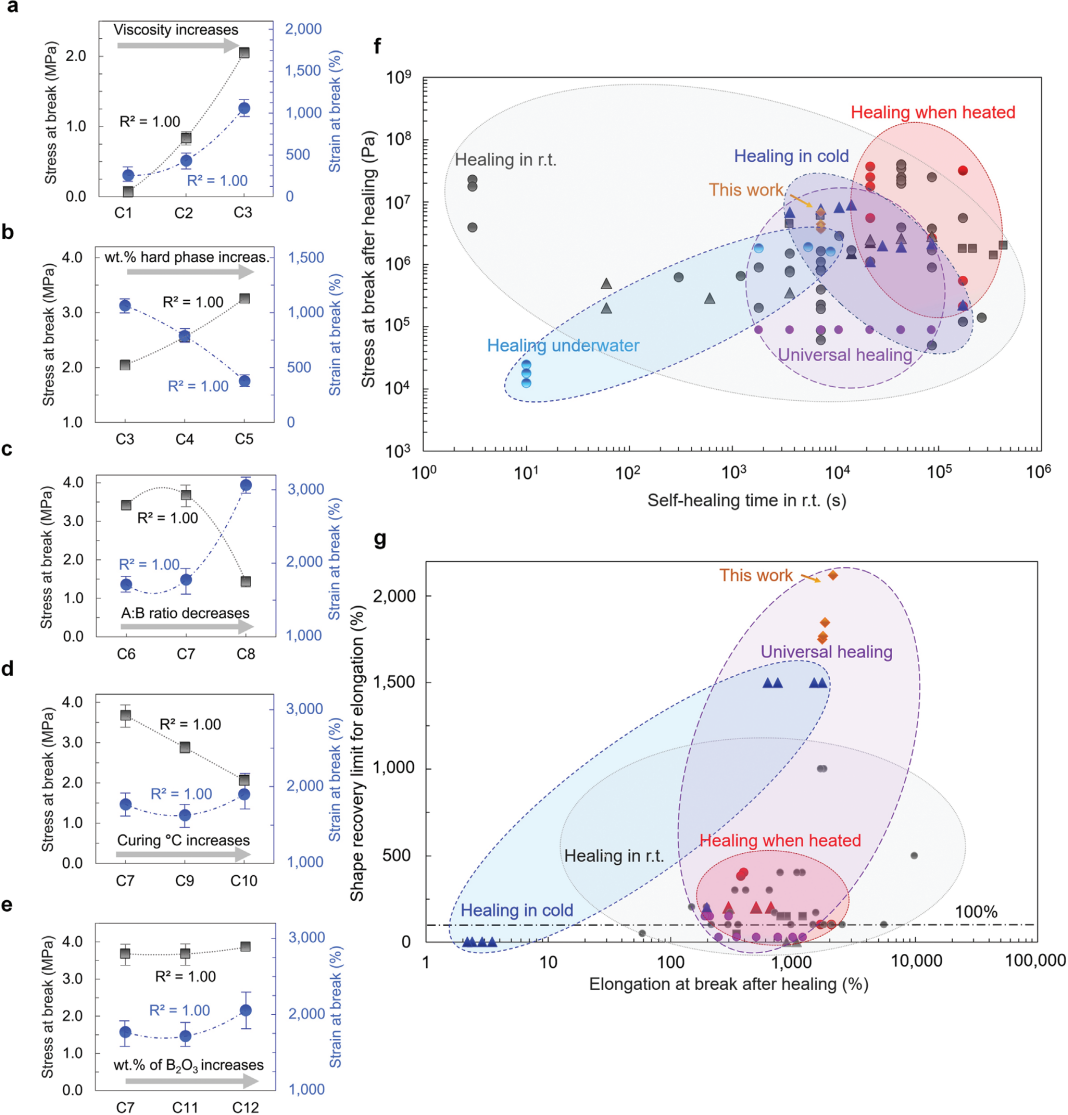
a–e) Stress at break and strain at break were plotted as a function of composition (detailed compositions are given in Table S1, Supporting Information). Data presented as mean ± minimum/maximum (*n* ≥ 3). a) Viscosity of PDMS‐OH increases from 850–1150 cSt to 18 000–22 000 cSt. b) Amount of hard phase increases from 15 to 25 wt%. c) Ratio of polymer base to cross‐linking component decreases from 10:1 to 2.5:1. d) Curing temperature increases from 70 to 150 °C. e) Amount of B_2_O_3_ NPs increases from 0.85 to 2.12 wt%. f,g) Performance of the elastomer is compared to state‐of‐the‐art self‐healing materials. Hydrogen bonds, metal–ligand interactions, and other mechanisms (ionic, combined, etc.) are denoted by circles, triangles, and squares, respectively. (Data point references can be found from Figure S7, Table S3, and Section S3, Supporting Information).

To understand the mechanics of the newly developed elastomer, we studied the adjusting of mechanical properties by varying its composition (**Figure** **2**a–e and Table S1, Supporting Information). A nearly linear stress–strain relationship was found when specimens were uniaxially elongated (Figures S4–S6 and Section S2, Supporting Information) that is a characteristic of purely elastic material. Furthermore, it was found that the elastomer became increasingly robust by increasing length of the long polymer chains in the soft phase. Young's modulus (*E*) and stress at break (*σ*_break_) were increased by over tenfold following the composition change (Figure 2a). Subsequently, *E* became in the range of 0.3–1.1 MPa depending on the composition (with a strain rate of 5% s^−1^) (Table S2, Supporting Information). Interestingly, it was found that by decreasing the curing temperature and the amount of polymer base (in hard phase), there was a twofold increase in *σ*_break_ and elongation at break (*ε*_break_) (Figure 2c). The improvement of mechanical properties could be a result of the existing microphase‐separated morphology between the soft and the hard phase with a particular composition (as discussed later on).

Mechanical properties were compared to other state‐of‐the‐art self‐healing materials (Figure 2f‐g, Figure S5 and Tables S3‐S5, Supporting Information). It was found that *σ*_break_ was up to two orders of magnitude larger than in the existing universal and underwater self‐healing materials. Introducing a multimodal chain length distribution to universal self‐healing materials is therefore an effective strategy to increase both the strength and the toughness by also facilitating strain‐induced reinforcement. In this case, the bimodal interpenetrated network consists of a small number of shorts chains (inverse to that with non‐healable rubber‐like networks)^[^ ^39^ ^]^ that are incorporated to the elastomer by the hard phase (provides structural integrity). The boron oxide nanoparticles have also reinforcing effect through following mechanisms. Chemisorption of boron oxide nanoparticles may increase effective degree of cross‐linking and reduce segmental mobility of the adsorbed polymers chains near Si‐O‐B‐O‐Si and O‐B bonds. Then, reinforcing filler particles can participate in the molecular rearrangements under strain through existing stress gradients and decreased chain mobilities of nearby bonds.^[^ ^39^ ^]^ Both of which can increase the stiffness of the network. Achieving a high strength and toughness with self‐healing materials is particularly important to increase mechanical damage resistance, and especially important in soft electronics as devices must be made thin enough. Low mechanical strength also poses other severe challenges, for instance, to achieve effective system scale self‐healing (with assumption of matched surface chemistries) due to improper alignment of multilayers.

Relaxation times were also measured by elongating samples to 100% and 500% strain (Figure S8 and Section S4, Supporting Information). The stress‐relaxation times for pristine elastomers were approximately in the range of 25–750 s. The elastomers showed excellent structural stability as considerable static loads could be endured for longer periods of time and the shape was recovered on removal of the load. The tested samples were indeed highly elastic as shown by the dynamic stretch‐release process (Movie S1, Supporting Information). However, they were not purely elastic as they also exhibited pronounced mechanical hysteresis (i.e., an ability to dissipate a significant amount of kinetic energy upon deformation). It was found that the composition with the most efficient shape recovery and the highest conformational entropy changes per unit area (please see discussion in Section S1, Supporting Information) exhibited also the lowest mechanical hysteresis (Figure S9, Supporting Information). It is well‐known that physiochemical interactions slow failure mechanisms (such as crack growth propagation), but they are also necessary in delaying an instantaneous rupture occurrence.^[^ ^20^, ^25^ ^]^ The ability to reversibly break and re‐form bonds is one of the reasons why self‐healing materials withstand large deformations without rupturing (but this may compromise structural stability under static load), whereas magnitude of their mechanical hysteresis may be related to level of intertwining of chain segments and molecular rearrangement under stress.

As the length of the polymer chains in the soft phase increases, both elastic and inelastic characteristics are present in its shape recovery. Interestingly, elastomer with a shorter chain length in soft phase had a near instantaneous response to strain with no creep and little or no stress relaxation (Figure S8, Supporting Information). When a specimen was elongated beyond its breaking point at a fast rate, it fractured into two pieces that rapidly recovered their shape (a result of entropy elasticity). Surprisingly, when elongating the elastomer to its *ε*_break_ (at slow rate of 5% s^−1^), the fractured pieces still gradually retracted to their original size. If any notches existed, they gradually decreased in size and eventually healed (Figure S10, Supporting Information). Unaligned individual pieces fitted perfectly together after retracting close to their original shape. Especially at slow rates, the weak dynamic interactions in self‐healing materials can often facilitate intermolecular slippage at large elongations leading to poor shape recovery on removal of stress (which is obviously is not the case here).

The bimodal elastomers also exhibited strain rate‐dependent characteristics, thus values of *E, σ*_break_, and *ε*_break_ depended on the level of elongation. Interestingly, we found that as the rate increased from 5 to 25% s^−1^, pristine C3 fractured with *σ*_break_ = 1 MPa at *ε*_break_ = 280% in comparison to *σ*_break_ = 2 MPa at *ε*_break_ = 1070%. Also, during even faster stretch‐release process (Movie S2, Supporting Information), the specimens were fractured on low deformation. It was assumed that this dilatant characteristic of pristine elastomer was a remnant of the non‐Newtonian substance forming the soft phase. As recently shown, the shear‐stiffening effect of the soft phase relates to insufficient dynamic relaxation of supramolecular interactions (Si‐O:B dative bonding and hydrogen bonding) at high strain rates. These bonds can form temporary crosslinking points as their connections lock their position.^[^ ^35^ ^]^

A strain hardening (i.e., a rapid increase of stress at certain levels of strain) was evident for most of the compositions due to bimodality of the interpenetrated network. The strain hardening had an increasing tendency and became more vicious as a function of strain level (Figures S4, S5, and S9, Supporting Information). A reversible photoelasticity effect was existed upon elongation as a result of orientation of long‐chain molecules (similar to that of rubber).^[^ ^34^ ^]^ This leads to birefringence as the level of crystallinity changed on the crystalline and amorphous regions which formed boundaries upon deformation. It is known that bimodality of the network facilitates strain‐induced reinforcement in nonhealable rubber‐like networks (which is also the case herein).^[^ ^39^ ^]^ It is expected that the forceful strain‐induced reinforcement and photoelastic effect in the polydimethylsiloxane‐based elastomer was a result of chemisorption of nanoparticles with further assumption that nonaffine deformation exists, orientation in the chain segments increases with molecular rearrangements, and segmental mobility is reduced near the filler surfaces (i.e., nanoparticles and short chains are redirecting tension in the network).

**Figure 3:**
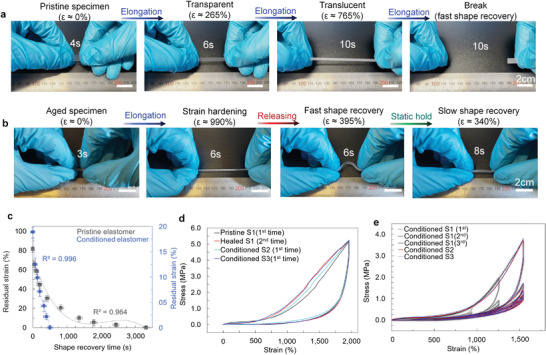
a) Photographs of photoelastic effect in pristine elastomer on rapid deformation when elongated to break (Movie S2, Supporting Information). b) Photographs of elastic response of a conditioned specimen (Movie S3, Supporting Information). c) Shape recovery times for pristine and conditioned specimens when load is removed after elongation to their *ε*_break_. Data presented as mean ± SD (*n* = 5). d) Stress–strain curves for specimens that were conditioned differently (further details in Figure S8, Supporting Information). e) Stress–strain curves during five consecutive cycles for conditioned elastomer specimens when elongated to 950%, 1250%, and 1500% with strain at rate 25% s^−1^.

The elastomers were 100% resilient up to their stretching limit and achieved perfect shape recoveries. Pristine specimens retracted to their original shape in ≈3300 ± 150 s (mean ± SD) after elongated to break (at slow rate of 5% s^−1^). A specimen could be instantaneously stretched to its limit after shape recovery (**Figure** **3**b,c). After healing, a specimen displayed remarkably similar mechanical properties compared to pristine samples. Elastomer could not only fully heal themselves (or even improve their properties) but achieved shape recovery efficiency and resilience was higher than in any existing self‐healing materials (Figure 2g). We hypothesize a reason for such strong entropic recovery of properties could relate to microphase‐separated interfacial regions between the soft and the hard phase (Section S1, Supporting Information). Then, the phase‐separated morphology could not only protect the soft phase^[^ ^40^ ^]^ but also provide stable junctions points (as shown with thermoplastic polycaprolactone–polyurethane fibers^[^ ^38^ ^]^) which would be especially important under large mechanical deformations.

The state‐of‐the‐art self‐healing materials can show considerable amount of plastic deformation even before elongation to their breaking point. Shape recovery may require very longtime scales (days, weeks, or even much longer than that), or else it is limited only to small deformations (i.e., poor resilience) due to the viscoplastic nature of the polymer networks (Figure 2g). Such a phenomenon has not only a detrimental effect on the dynamics of soft materials but would render their smart skins inaccurate and unreliable. The poor shape recovery can be understood, e.g., through a simplistic Kelvin–Voigt model where entropic springs reach their equilibrium very slowly. Viscoplasticity and poor resilience arise from occasions when an entropic spring irreversibly breaks at critical stress leading to an absence of a restoring force (resulting in intermolecular slippage and flow in a 3D network).

To better understand the complexity observed in the bimodal elastomers’ mechanical and self‐healing properties, and their dependency on the predisposed conditions (such as stress–strain history), the mechanical hysteresis was studied in more detail (Figure S11 and Section S5, Supporting Information). It was found that the elastomer could be brought into stable state in numerous ways, although some of them proved better than others. These conditioned specimens were very different from pristine ones (Figure 3a–c) as they could be adapted to withstand very rapid elongations (rate of 330% s^−1^) up to very large deformations (≈1000% strain) without fracturing (Figure 3b and Movie S3, Supporting Information).

More impressively, conditioned specimens had over an order of magnitude faster shape recovery times (Figure 3c) and could recover their shape with a rate ≈660% s^−1^ in the strain hardening region (Figure 3b). Further evidence of their elastic characters becoming more dominant was visible changes in the stress‐relaxation times (Figure S7, Supporting Information). Because relaxation times increased with conditioning, they can adapt to withstand larger elongation with faster strain rate without rupturing (as critical relaxation time is not exceeded). Thus, structural integrity and stability of the bimodal network increases with mechanical conditioning (please see further discussion in the Supporting Information). In future, an appropriate viscoelastic model for the new material system could be derived by postulating a Schapery‐type viscoelastic model including viscoelastic components for Helmholtz free energy and rate of energy dissipation, and entropy imbalance (known as the Clausius–Duhem inequality).^[^ ^41^ ^]^ We point out that entropy‐driven elasticity is also a valid model to account for a possible reversible phase transition occurring on large deformation, having particular importance in this case.

### 2.4 Universal Self‐Healing Properties

**Figure 4:**
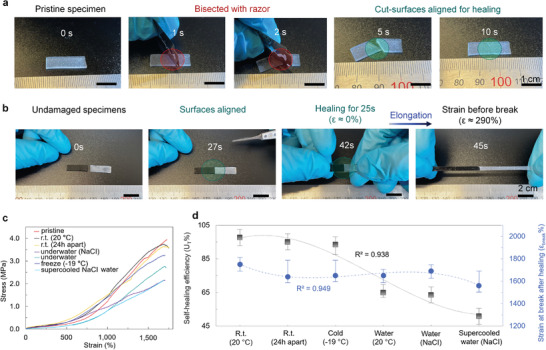
Self‐healing properties of elastomers. a) Photographs of specimen under test when cutting diagonally and perpendicularly to direction of uniaxial elongation. b) Photographs of specimens when cut‐surfaces are aligned and interface is healed at room temperature before elongation. The first‐rate healing capabilities are evident from the experimental results as the specimens could be elongated to 290 ± 50% strain with rate of 60–100% s^−1^ after healing for 23.6 ± 5.5 s. c) Stress–strain curves for pristine and healed specimens when elongated to *ε*_break_. d) Self‐healing efficiency for toughness and strain at break after healing for specimens in various ambient conditions. Data presented as mean ± minimum/maximum (*n* ≥ 3). (The elastomer compositions were C7.)

The universal self‐healing properties of the elastomers were studied under different conditions (**Figure** **4**a–d). It was assumed that effective amount of boron oxide nanoparticles was present in the composition's due presence of strong dynamic interactions (Table S2 and Figure S2, Supporting Information). Specimens with the most efficient shape recovery appeared to have also the most efficient self‐healing (*η* = 97.6 ± 4.8% (mean ± SD)). Moreover, the time scales for self‐healing became much more reasonable for practical applications (up to orders of magnitude smaller), and self‐healing efficiencies could be higher than in existing self‐healing materials.^[^ ^13^, ^17^, ^21^, ^27^, ^29^ ^]^ However, the lack of technical testing standards may hinder comparison with the different self‐healing materials.

We found no sign of surface aging effects as in many cases with only hydrogen bonding. To prove this, two undamaged specimens were successfully permanently self‐bonded together (Figure S12 and Movie S4, Supporting Information). The interface remained intact after being elongated to break after only ≈20 s of healing. Subsequently, as cut‐surfaces were kept apart for ≈24 h and then aligned for healing, high self‐healing efficiencies were achieved (*η* = 95.0 ± 4.8%) (Figure 4d). This relates to the O—B bonds that can be continuously in brink of equilibrium (i.e., cannot be saturated) when in hydrated state in the presence of water molecules.

The elastomers were stable in high‐humid conditions and even underwater. When specimens were kept underwater for extended period of time (up to 3 days), their dimensions did not visibly change, and the weight was increased by ≈1.5–3.5% underwater that was dependent on the ambient conditions (Figure S13, Supporting Information). Elastomers tend to float in an aqueous medium unless they self‐adhere to solid surfaces or are forcefully kept under water due to surface tension and hydrophobicity (Figures S14 and S15, Supporting Information). Underwater bisected and aligned pristine specimen could be immediately elongated to ≈150% strain when healed for ≈30 s under water (Figure S14, Supporting Information).

Temporary softening of elastomers can be explained through Coulomb interaction and the high relative permittivity of water.^[^ ^42^ ^]^ As electrostatic force is inversely proportional to the dielectric constant (stated by Coulomb's law *F* = (*q*_1_ *q*_2_ $\hat{r}$_12_)/(4*πε*_0_∙|*r*_12_|^2^)), the mechanical robustness in a hydrated medium decreases with increase of relative permittivity. Elastomers reverted to their original state from the temporary softened state as they dehydrated in air thus regaining their entropy level before hydration. Even though, the elastomers achieved swelling equilibrium fast under water, deswelling can take significant amount of time without external stimuli.

The self‐healing efficiencies in 26.3 wt% of sodium chloride (NaCI) water (*η* = 63.5 ± 4.8%) were similar to that when healed in pure water (*η* = 65.0 ± 2.5%) (Figure 4c,d). Self‐healing efficiency would be expected to be higher in saline water as dielectric constant of the water decreases with salinity content (explained by the change in the orientational polarization).^[^ ^43^, ^44^ ^]^ Then, polymer medium should benefit as a larger electrostatic force of attraction can exist between two oppositely charged point charges (but sample size was relatively small). It is important to address the effect of softening as it impairs load bearing capability and decreases mechanical damage resistance compared to the nonsoftened case. Herein, the reversible softening has controversial effect as it increases the self‐healing rate at the expense of mechanical performance (as shown later on).

To demonstrate the self‐healing could be maintained in both dry and wet conditions in low temperatures, the elastomers were first kept in freezer for over 2 h (−19 °C). The elastomers achieved high self‐healing efficiency (*η* = 93.4 ± 5.4%) similar to that at room temperature (Figure 4d). It is well known that a low glass transition temperature (*T*_g_) allows maintaining high chain mobility and fast diffusion of dynamic bonds to damaged locations even in extremely low temperatures. However, as previously shown, boron modifications may increase the *T*_g_ of the elastomer close to −100 °C (in comparison to poly(dimethylsiloxane) with *T*_g_ in the range of −120 to −150 °C).^[^ ^36^ ^]^ To validate the self‐healing efficiency under more harsh environments, the self‐healing functionality was tested in supercooled saline water (−19 °C). Self‐healing efficiencies as high as *η* = 50.8 ± 4.7% were maintained as elastomers healed in supercooled NaCI water (26.3 wt%) for 2 h. The reason for decreased self‐healing efficiency could be as simplistic as the diffusion of dynamic bonds becomes slower as supercooled water molecules are adsorbed into the polymer medium. Further investigations are still needed and especially under more extreme environments.

### 2.5 Universal Self‐Healing Mechanism in Dry and Wet Conditions

**Figure 5:**
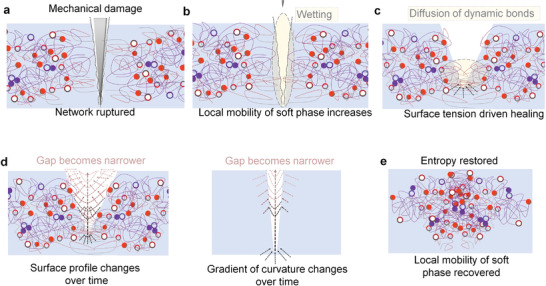
Universal self‐healing mechanisms in dry conditions. a–e) Schematic illustration of the mechanism in air without intervention (when the pieces are not placed together after the damage). The self‐healing process is both surface tension and entropy‐driven. a) Conformational entropy decreases upon deformation leading to b) increased local mobility and wetting by polymer. c) Surface tension‐driven healing leads to diffusion of dynamic bonds at bottom of the cut. d) Surface profile changes over time as result of surface tension and entropy‐driven shape recovery. Wound closure follows as the gradient of curvatures increases. e) Conformational entropy of the system and strength of the polymer become fully recovered with randomization as free energy is minimized.

The stages of self‐healing in dry conditions with the elastomers are similar to the well‐known five stages of crack healing.^[^ ^45^ ^]^ The self‐healing without intervention in the bimodal elastomer network is driven by surface tension after wetting (Movie S6, Supporting Information) leading to the gradient of curvature decreasing with the diffusion of O—H and B—O bonds over the damaged area (**Figure** **5**a–e). Transition to entropy‐driven healing may occur once the surface tension decreases as a consequence of reduced damaged areas and shape recovery follows. As dynamic bonds have diffused to their equilibrium distances, conformational entropy and strength of the bimodal elastomer network become restored with randomization. Then, bimodal elastomer recovers its permanent shape that was fixed during cross‐linking.

**Figure 6:**
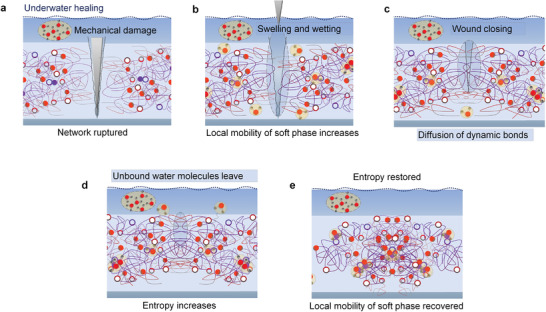
Universal self‐healing mechanisms in wet conditions. a–e) Schematic illustration of the self‐healing mechanism under water without intervention. a) The mechanical damage underwater in the swollen elastomer b) leads to more entropy‐driven healing mechanism and faster wound closures (as elastomer minimizes free energy by retracting). c,d) This is beneficial for wetting by polymer and diffusion of dynamic bonds in damaged areas. e) The entropy and strength of the material are gradually restored as dynamic bonds diffuse to their equilibrium distances and network becomes randomized.

The surface of pristine elastomers may self‐heal even faster without intervention in wet conditions than in dry conditions (Movie S6, Supporting Information) that can be accounted for due to multiple factors (please see further discussion in Section S6, Supporting Information). For instance, any degree of swelling can facilitate faster wetting and wound closure (**Figure** **6**a–e) and it is evident that hydrophobic domains play a key role.^[^ ^17^, ^21^, ^23^ ^]^ The entropy and amount of free energy of the system changes underwater on deformation as liquid content changes, and after swelling equilibrium is achieved. The latter is dependent on the polymer's interaction with water molecules. Herein, the condensation/hydrolysis equilibrium can be achieved fast due to nature of the Si‐O:B dative bonding being highly sensitivity to the presence of water molecules and solvents. Because water–polymer interactions are unfavorable within hydrophobic domains^[^ ^25^, ^41^ ^]^ and in the end water is energetically unfavorable for the free energy of a system, unbound water molecules are eventually removed from the system underwater. Another reason for such excellent underwater healing capabilities relates to the O—B bond as previously demonstrated.^[^ ^46^ ^]^ Reversible condensation/hydrolysis equilibrium (‐B(OH)_2_ ⇌ B‐O‐B+H_2_O) increases the supramolecular interaction of Si‐O:B dative bonding also as ambient humidity increases (because additional free functional groups may appear).^[^ ^36^ ^]^

## 3 Application as Mechanochromic Sensor

It is known that the periodic nano‐ and microstructures found in nature create the most vivid and iridescent colors.^[^ ^47^, ^48^ ^]^ Some species can adjust color on stimulus or when “triggered” through optical interference effects—as synthetic materials with periodic assemblies.^[^ ^49^, ^50^, ^51^ ^]^ For instance, a hydroxypropyl cellulose (HPC) self‐organizes into cholesteric phase on critical concentration in solvents.^[^ ^47^, ^52^, ^53^ ^]^ The cholesteric phase is known to consist of rod‐like molecules in an arrangement of periodic helical order. The pitch along the helix axis changes with stimuli (stress, humidity, or temperature) giving rise to a Bragg‐like reflection of circularly polarized light at a specific wavelength.

**Figure 7:**
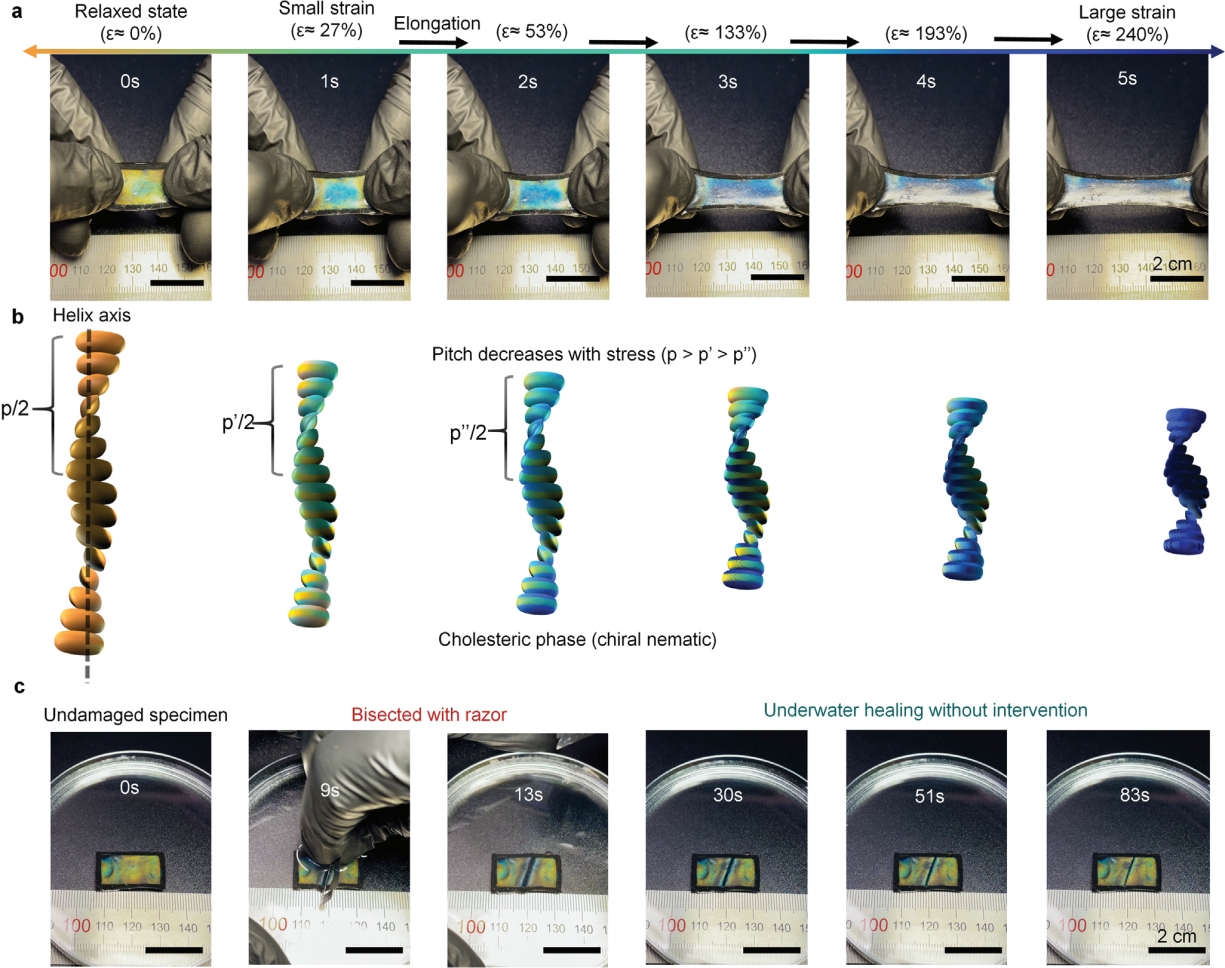
a,b) Photographs of color responses under uniaxial tensile stress and schematic illustration of the working mechanism. b) The pitch (*p*) of the cholesteric phase gives the distance along the helix axis. The pitch is determined from the De Vries’ formula *λ*_0_ *=* (*n*_o_ *+n*_e_)*p*/2, where *λ*_0_ is the wavelength at the center of the reflection band, *n*_o_ and *n*_e_ are the average refractive indexes of the nematic planes.^[^ ^58^, ^59^ ^]^ Maximum reflectance is approximated through Bragg's law (*λ = np*cos*θ*), where *n* is the average refractive index of the cholesteric phase and *θ* is the angle between the incident light and the helix axis. Upon deformation, the wavelength of the reflected light shifts from ≈600 to 450 nm enabling a visible and reversible color shift to occur. c) Photographs of underwater self‐healing without intervention after mechanochromic sensor is bisected (Movie S8, Supporting Information).

Even though, self‐healing liquid metal‐based conductors have been achieved in combination with elastomers, self‐healing of mechanochromic liquid phases has not been shown (when not cross‐linked with polymers).^[^ ^21^, ^54^, ^55^, ^56^, ^57^ ^]^ Herein, a universal self‐healing mechanochromic sensor (**Figure** **7**a–c) was prepared that may have considerable advantages over its counterparts with electrical transducing modes (please see further discussion in Section S7, Supporting Information). The sensor showed a reversible and vivid color shift with elongating stimuli (Movie S7, Supporting Information). The color was saturated to dark blue when elongated to ≈300% strain which was hardly visible as the substrate had a reverse photoelastic effect (transparency increases with strain). The sensor could recover its functionality even after suffering fatal damages (Figures S15 and S16, Supporting Information) in various ambient conditions and even without intervention (Figure 7c and Movie S8, Supporting Information). Interestingly, the mechanochromic effect of the cholesteric phase was revealed in real‐time surface tension near the damaged location as the sensor self‐healed and recovered its shape (this corresponded to that seen with optical microscopy). Even though neither the design nor the mesophase was not yet optimized for this kind of sensor, it already showed promising sensing and self‐healing characteristics. It is the intention to optimize the design in the future to explore the sensing and self‐healing capabilities in detail.

## 4 Conclusions

In the past, a major obstacle has been to achieve both high mechanical and self‐healing performance in a single universal self‐healing material. Extensive developments in the field of self‐healing materials and their applications are still needed before they are widely applicable to industry. However, as the findings show, bimodal interpenetrated network with entropy‐driven elasticity is a viable strategy to achieve mechanical robust and highly resilient universal self‐healing materials. We also discovered an unprecedented behavior that allowed a conditioning process and further improvement of mechanical properties of the material. It is expected that understanding this phenomenon may lead to significant discoveries in the future. One of the potential application areas could be a universal self‐healing photonic smart skin for various applications, as demonstrated in this paper.

## 5 Experimental Section

### Materials

Hydroxyl terminated poly(dimethylsiloxane) (PDMS‐OH) with kinematic viscosities of 850–1150 cSt and 18 000–22 000 cSt and HPC (Mw ∼ 100 000) were purchased from Sigma‐Aldrich. Boron oxide nanoparticles (B_2_O_3_, 80 nm) were purchased from SkySpring Nanomaterials Inc. Carbon black powder (Vultan XC72) was received from Cabot Corporation. Dimethylvinyl‐terminated dimethylsiloxane‐based elastomer was purchased from Sil‐mid Limited. All materials were used as received.

### Synthesis of Self‐Healing Elastomers

≈0.8–2.2 wt% of B_2_O_3_ nanoparticles were mixed in a mortar with dimethylvinyl‐terminated dimethylsiloxane‐based elastomer. Then PDMS‐OH with viscosities of 850–1150 and/or 18 000–22 000 cSt was added to the solution. The solution was vigorously mixed at room temperature before continuing the mixing with a magnetic stirrer. The solution was vacuum degassed to release the entrapped voids and was then drop cast onto a petri dish or onto a glass substrate with a release film. The cross‐linking of the elastomers was carried out at elevated temperatures of 70–150 °C. After peeling the elastomer from the release film, samples were cooled at room temperature for at least 24 h before any measurements were taken.

### Preparation of Self‐Healing Mechanochromic Sensor

≈60 wt% of HPC (Mw ∼ 100 000) was vigorously mixed with deionized water. The solution was repeatedly mixed and kept sealed in a glass vial. To avoid undesirable optical coupling from a substrate, ≈1 wt% of a carbon black powder (Vultan XC72) was mixed into the PDMS‐OH base. Transparent self‐healing elastomer and carbon‐filled self‐healing elastomer substrate were solution‐cast onto release films. The HPC 60 wt% solution was stencil printed onto a substrate with a rectangular cavity. Multilayers consisting of a transparent elastomer, HPC, and substrate were constructed, and transparent elastomer film was pressed against the substrate to self‐adhere the layers together.

### Morphology Characterization

FTIR spectra from the elastomers were measured with a Thermo Scientific Nicolet iS5 FTIR spectrometer with D7 Diamond ATR. Optical imaging and video recordings were taken with an Olympus BX51 microscope with U‐POTP3 polarizer.

### Thermal Characterization

TGA and DSC were conducted with NETZSCH STA 449F3 Jupiter for elastomers over temperature range of 30–550 °C in oxygen atmosphere with a heating rate of 10 °C min^−1^ (measurements were calibrated with reference).

### Mechanical Characterization

Mechanical properties were characterized by performing stress–strain tests with a Linkman TST350 Tensile Stress Testing system with a 200 N force transducer at room temperature (20 °C). Rectangular samples had dimensions ≈25.0 mm x 7.0 mm x 1.5 mm (length x width x thickness). Since the difference between a nominal stress and true stress was considerable, the true stress was calculated based on the nominal stress with the assumption that the elastomer was isotropic and incompressible (i.e., its volume did not change with compression). The true cross‐sectional area calculated was found to agree approximately with the experimental results. The distance between clamps was fixed to ≈2.5–5.0 mm (depending on composition). Each measurement was repeated at least three times and at least ten samples were measured from each composition. Unless otherwise stated, all mechanical characterizations were performed at a rate of 5% s^−1^.

### Self‐Healing Characterization

Self‐healing efficiencies for elastomers were defined as the proportion of toughness recovered relative to the original toughness of a pristine elastomer since it did not only take into account the stress at break (*σ*_break_) but also the elongation at break (*ε*_break_). This was corresponded to the area under the stress–strain curve which defined a material's ability to absorb energy until fracturing. Unless otherwise stated, the self‐healing tests were conducted by creating a diagonal cut with a razor that was perpendicular to the direction of elongation (Figure 4a). The dissociation areas were 100% of the cross‐sectional area of the sample under test. Unless otherwise stated, cut surfaces were aligned within ≈10 s after samples were bisected (in all cases). However, when surface ageing effects were tested, the cut surfaces were kept apart for 24 h at room temperature. C1 and C2 elastomers were not entirely separated (dissociation area > 90%) to properly align cut‐surfaces due to the softness of the materials.

### Statistical Analysis

Continuous variables were expressed as mean ± minimum/maximum (sample size (*n*), *n* ≥ 3), mean ± SD (*n* = 5), and mean ± minimum/maximum (*n* ≥ 3) for Figures 2a–e (fitted with a third degree polynominal function), Figure 3c (fitted with a cubic function), and Figure 4 (fitted with a cubic function), respectively. No statistical tests were used nor any softwares were used for the analysis of significance.

## Conflict of Interest

All authors have issued patent applications (EP 21172728.4 and EP 21172721.9) related to processes, materials, and devices described in this article.

## Supporting information

Supporting Information

Supplemental Video 1

Supplemental Video 2

Supplemental Video 3

Supplemental Video 4

Supplemental Video 5

Supplemental Video 6

Supplemental Video 7

Supplemental Video 8

## Data Availability

Research data are not shared.
