# Supplementary material for: All‐Around Universal and Photoelastic Self‐Healing Elastomer with High Toughness and Resilience
Source: Adv Sci (Weinh). 2021 Oct 18;8(24):2103235. doi: 10.1002/advs.202103235 (PMC8693070; doi:10.1002/advs.202103235)
Supplement: Supplementary file 1 — Supporting Information [file ADVS-8-2103235-s009.pdf]

© 2021 Wiley-VCH GmbH

ADVANCED SCIENCE
Open Access

Supporting Information

for *Adv. Sci.*, DOI: 10.1002/advs.202103235

All-Around Universal and Photoelastic Self-Healing Elastomer with High Toughness and Resilience

*Mikko Nelo, Jari Hannu, Jari Juuti, Heli Jantunen, and Jarkko Tolvanen**

Supporting Information

All-Around Universal and Photoelastic Self-Healing Elastomer with High Toughness and Resilience

Jarkko Tolvanen, Mikko Nelo, Jari Hannu, Jari Juuti, Heli Jantunen*

Dr. Jarkko Tolvanen*, Dr. Mikko Nelo, Dr. Jari Hannu, Dr. Jari Juuti, Prof. Heli Jantunen,

Microelectronics Research Unit, Faculty of Information Technology and Electrical Engineering, University of Oulu, P.O. Box 4500, FIN-90014 Oulu, Finland

E-mail: jarkko.tolvanen@oulu.fi

S1. Thermodynamic system, entropic-recovery, and entropy of a system

A thermodynamic system can be described by its enthalpy known as the thermodynamic potential or Gibbs free energy. The Gibbs free energy (*G*) for a thermodynamic system can be described by **Equation S1**.

$$G = U - TS + PV = A + PV \tag{S1}$$

where: *U* is internal energy, *T* is temperature, *S* is entropy, *P* is pressure, *V* is volume of a system, and *A* is the Helmholtz free energy.

In an isothermal (where temperature remains constant) and reversible process, Supplementary Equation 1 reduces to **Equation S2**. As the change in the Helmholtz free energy is directly proportional to the work done by an external force it can be written as below.

$$\Delta A = \Delta U - T\Delta S = -T\Delta S \text{ (when } \Delta U = 0) \tag{S2}$$

The conformational entropy becomes negative as the number of available chain conformations and the degree of freedom decreases when a polymer is deformed above its glass temperature (T_g) by an external force.

With the assumptions that the elongation is uniaxial and the material being incompressible (no change in the volume), the change of entropy (per unit of volume) is expressed by **Equation S3**.

$$\Delta S = -\frac{NkT}{2}\left(\lambda^2 + \frac{2}{\lambda} - 3\right) = -\frac{pRT}{2M_j}\left(\lambda^2 + \frac{2}{\lambda} - 3\right) = -\frac{v_jRT}{2}\left(\lambda^2 + \frac{2}{\lambda} - 3\right) \tag{S3}$$

where: *N* is Avogadro number, *k* is Boltzmann constant, *λ* is extension ratio (L/L_0) along elongation, *R* is universal gas constant, ρ is density, M_j is 'molecular weight' between junctions, and v_j ($v_j = p/M_j$) is junction density.

The force (per unit of cross-sectional area) can be calculated from **Equation S4**.

$$\frac{F}{A} = \sigma = v_jRT\left(\lambda - \frac{1}{\lambda^2}\right) \tag{S4}$$

where: F is applied external force, A is cross-sectional area of the specimen, and σ is true stress.

The shear modulus (*G*) for an incompressible material can be calculated from **Equation S5**.

$$G = \frac{E}{2(1+v)} = \mathrm{N}k\mathrm{T} = \frac{\rho RT}{\mathrm{M_j}} = \mathrm{v_j}RT \tag{S5}$$

where: *E* is elastic modulus and *v* is Poisson's ratio (that is 0.5 in this case).

It was calculated that the elastomer composition with the most efficient shape recovery had the highest conformational entropy change per unit of volume (up to -220 kJ/m^3) and junction density of ~80 mol/m^3. The junction density further increased with the amount of boron oxide nanoparticles (when taking into account the porosity in the specimens). We supposed that for entropic-recovery of properties in self-healing elastomers (high resilience, etc.), a high molecular weight polymer, sufficient junction density, and multiphase-separated morphologies are likely some of the key requirements. Also, we further hypothesize that an existing microphase separations between the soft and hard phase could indeed act as stable junctions preventing any intermolecular slippage and flow in the interpenetrated network (which would be especially important at large mechanical deformations). However, the importance of the phase-separated morphology is open for an argument due to lack of further evidence in the present work. Further aspects of the phase-separated morphologies and possible reversible phase-transitions will be studied in the future work.

As it is known, swelling in an aqueous medium is primarily related to the change of conformational entropy associated with the diffusion of liquid molecules into the polymer. The equilibrium degree of swelling (determined by the value of υ_2) can be established as the Gibbs free energy for further absorption of aqueous medium is zero ($\delta G/\delta n_1 = 0$). In this case, n_1 is number of moles of liquid in the swollen polymer and υ_2 is volume fraction of polymer in the mixture. The term $\delta G/\delta n_1$ (also known as the molar free energy of dilution) can be defined as a sum of free energy mixing (G_m) and elastic deformation (G_e). Then, the term can be expressed by **Equation S6**.

$$\frac{\delta G}{\delta n_1} = \frac{\delta G_m}{\delta n_1} + \frac{\delta G_e}{\delta n_1} = RT\, ln(1-\upsilon_2) + \upsilon_2 + \chi\upsilon_2^2 + \frac{pV_1}{M_j}\upsilon_2^{\frac{1}{3}}) \qquad \text{(S6)}$$

where: χ is a constant specific to the system under observation (defining interaction between polymer and liquid) and V_1 is a molar volume (that is a molar mass divided by mass density) of the swelling liquid.

Then, the work done by deformation (associated with conformational entropy change (per unit of volume)) in a swollen material (with the assumption of pure homogenous strain and incompressibility) can be calculated from **Equation S7**.

$$\Delta S = -\frac{NkT}{2}\upsilon_2^{\frac{1}{3}}(\lambda^2 + \frac{2}{\lambda} - 3) \qquad \text{(S7)}$$

References for this section:

1. Theloar, L.R.G., The elasticity and related properties of rubbers. Rep. Prog. Phys., vol. 36, p. 755-826 (1973). https://doi.org/10.1088/0034-4885/36/7/001

2. Huber, J.M., Theory of the Elastic Properties of Rubber. J. Chem. Phys., 11, 455 (1943). https://doi.org/10.1063/1.1723785

3. Hiemenz, P.C., Lodge, T.P., Polymer chemistry. CRC Press, Boca Raton (2007)

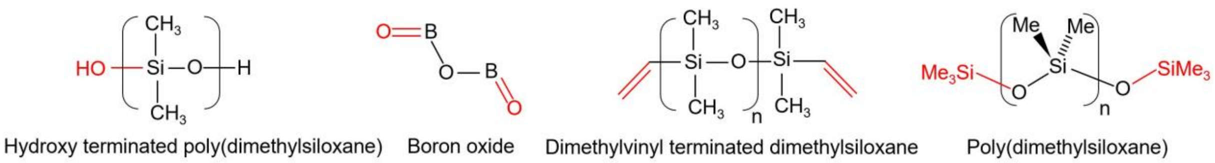

Figure S1. Chemical structures for hydroxy terminated poly(dimethylsiloxane), boron oxide, dimethylvinyl terminated dimethylsiloxane, and poly(dimethylsiloxane).

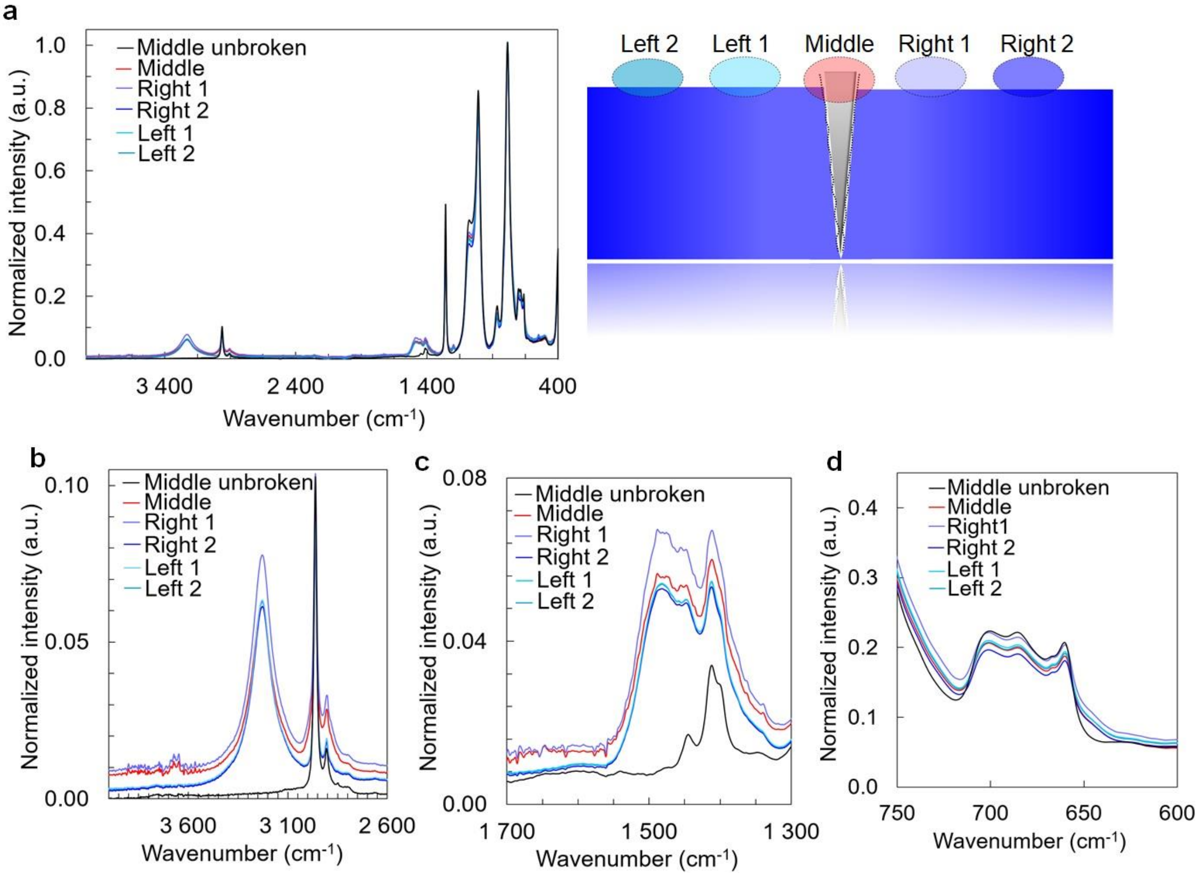

Figure S2. a) Fourier transform infrared (F-TIR) spectra for C3 pristine elastomer in static state when applying a small force as the probe tip was in physical contact with the specimen. To test the dynamic state, the specimen was damaged before the probe tip was placed in physical contact with the surface. The F-TIR spectre with other compositions was similar when the kinematic viscosity of the hydroxy terminated poly(dimethylsiloxane) was high.

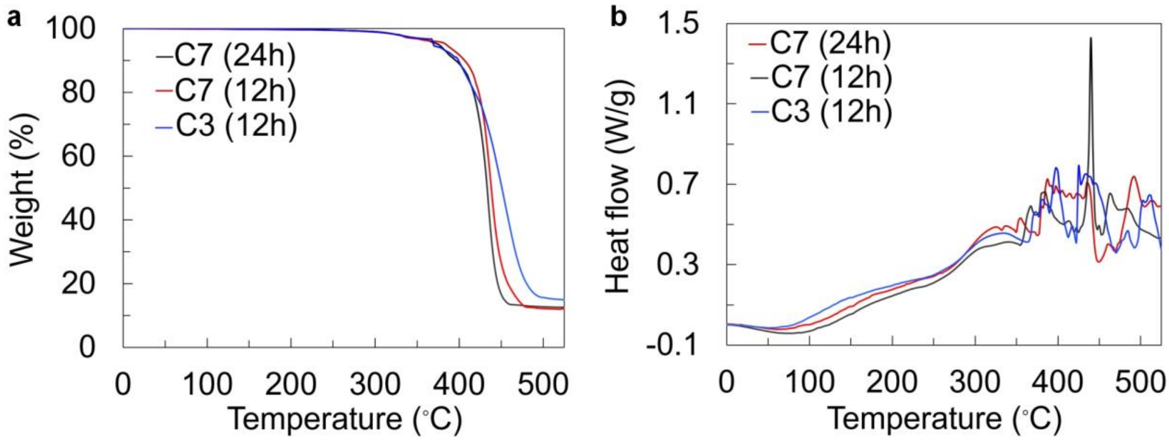

Figure S3. a) Thermogravimetric analysis (TGA) and b) differential scanning calorimetry (DSC) for elastomers at heating rate of 10 °Cmin^{-1}. After eliminating the thermal history, the data was collected from the second heating process. The cross-linking time for the elastomers C7 and C3 was expressed in the parentheses.

S2. Mechanical properties for elastomer compositions

Mechanical and self-healing properties were measured at room temperature with rate of 5%s^{-1} (**Figure S4 and S5**). The specimens were bisected and manually put together following damage (details in the Methods-section). Detailed composition parameters can be found in **Table S1**.

The number of effective cross-links increases with the end-to-end distance of the long polymer chains (in the soft phase) (**Figure S5a-c**). This significantly contributes to the overall strength of the network and facilitates a strain-induced reinforcement in the bimodal network (with the combination of short chains). When changing the bimodal chain length distribution by increasing the number of short chains (**Figure S4d-f**), there was significant increase in the upturn of the modulus and the elongation at which it begins decreased by over two-fold (from 380% to 150% strain).

By increasing number of covalent bonds (i.e., permanent net-/junctions points) with amount of cross-linking component (in hard phase), only the elongation required for the upturn in the modulus increased (from 380% to 1000% strain). The bimodal network simultaneously becomes more stretchable but shows poor shape recovery and resilience. When uniaxially elongated by 500%, a residual strain more than 50% remained after several days of resting at room temperature (indicating intermolecular slippage and flow in the network).

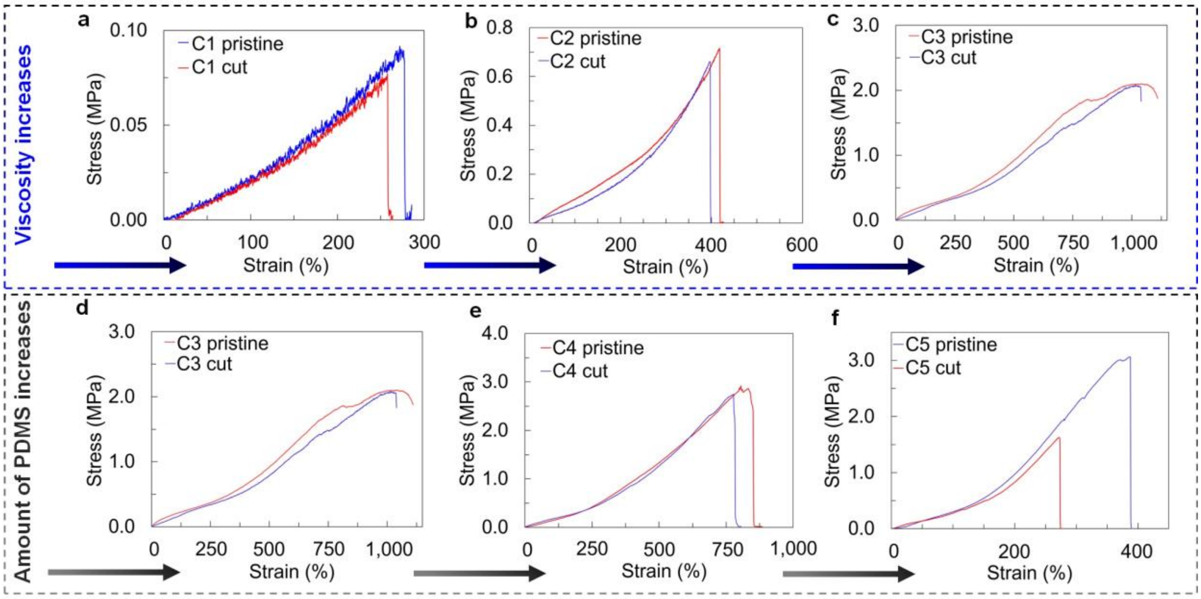

Figure S4. Stress-strain curves for pristine and healed elastomers with different composition. a-c) viscosity of PDMS-OH increases from 850-1,150 cSt to 18,000-22,000 cSt. d-f) amount of hard phase increases from 15 to 25 wt.%. Stress-strain curves for healed samples were recorded after healing for ~2 hours at room temperature (20 °C).

The strain-induced reinforcement is hindered by decreasing junction density as the curing temperature increased (which relates to the lower degree of chemisorption between boron oxide nanoparticles and polymer) (**Figure S5d-f**). This decreases the overall mechanical performance of the elastomer while its self-healing properties neither increase (**Table S2**). As seen, the amount of boron oxide nanoparticles did not increase the strain-induced reinforcement or mechanical robustness (**Figure S5g-i**) only because porosity of the specimens simultaneously increased.

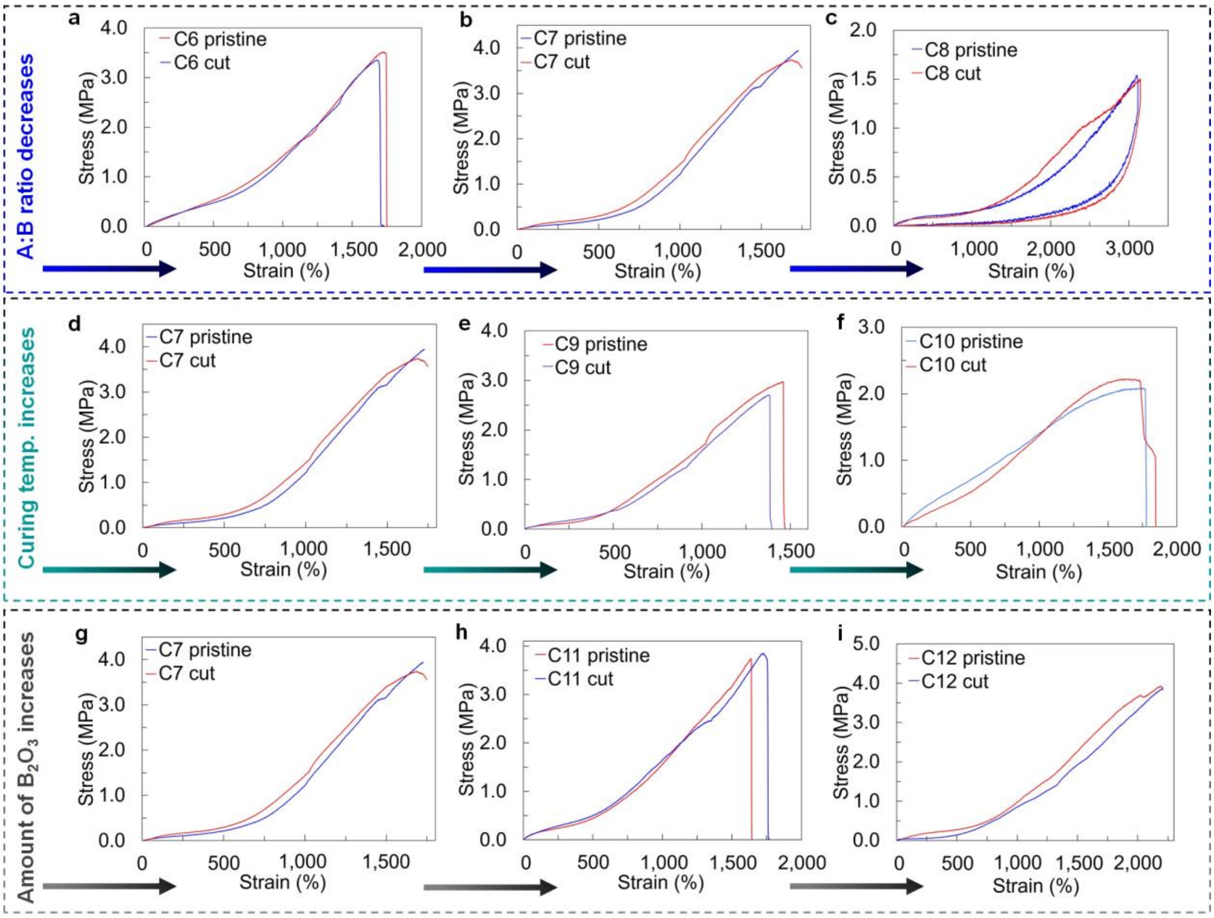

Figure S5. Stress-strain curves for pristine and healed elastomers with different composition. a-c) The ratio of polymer base to cross-linking component decreases from 10:1 to 2.5:1. d-f) The curing temperature increases to 70 °C and 150 °C. g-i) Amount of B_2O_3 NPs increases from 0.85 to 2.14 wt.%.

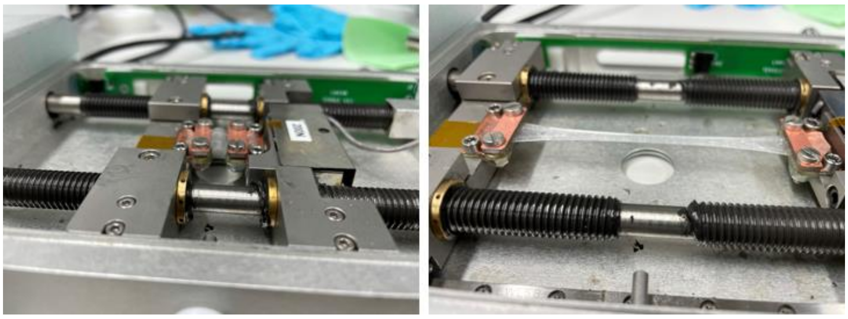

Figure S6. Photographs of tensile testing system and a specimen under elongation. The distance between clamps was adjusted depending on the composition (applied strain ~1,600% and rate 5%s^{-1} in these particular photographs).

Table S1. Elastomer compositions. Kinematic viscosity of hydroxyl-terminated poly(dimethylsiloxane)(PDMS-OH), weight percent (wt.%) of hard phase, ratio of polymer base to cross-linking component, curing temperature (°C), and wt.% of boron oxide nanoparticles (B_2O_3 NPs) were varied.

**Composition name**	**Viscosity of PDMS-OH (cSt)**	**Amount hard phase (wt.%)**	**Ratio of polymer base to cross-linking component**	**Curing temperature (°C)**	**Amount of B_2O_3 NPs (wt.%)**
C1†	850-1150	15.0	10:1	120	0.85
C2‡	850-1150/18,000-22,000	15.0	10:1	120	0.85
C3§	18,000-22,000	15.0	10:1	120	0.85
C4§	18,000-22,000	20.0	10:1	120	0.85
C-5§	18,000-22,000	25.0	10:1	120	0.85
C6§	18,000-22,000	15.0	10:1	70	0.85
C7§	18,000-22,000	15.0	5:1	70	0.85
C8§	18,000-22,000	15.0	2.5:1	70	0.85
C9§	18,000-22,000	15.0	5:1	120	0.85
C10§	18,000-22,000	15.0	5:1	150	0.85
C11§	18,000-22,000	15.0	5:1	70	1.49
C12§	18,000-22,000	15.0	5:1	70	2.12

† = PDMS-OH viscosity 850-1150 cSt (100 wt.%); ‡ = PDMS-OH with viscosities of 850-1150 cSt and 18,000-22,000 cSt mixed with ratio 1:1; § = PDMS-OH viscosity 18,000-22,000 cSt (100 wt.%);

Table S2. Elastic modulus (MPa), toughness (MJm^{-3}), strain at break (%), stress at break (MPa), elastic recovery, and self-healing efficiency for elastomer compositions. The properties were measured at room temperature (20 °C) with rate of $5\%s^{-1}$. Results are expressed as a mean value ± SD (n = 5).

Sample	Mechanical properties					Self-healing properties (after 2 hours in r.t.)	
	Young's modulus (MPa)	Toughness (MJ/m^3)	Strain at break (%)	Stress at break (MPa)	Elastic recovery	% recovery of toughness	% recover of strain at break
C1	0.04 ± 0.00	0.09 ± 0.01	261 ± 21	0.08 ± 0.01	No residual strain	82.5 ± 15.0	96.9 ± 0.8
C2	0.37 ± 0.00	1.30 ± 0.02	435 ± 31	0.84 ±0.01	No residual strain	84.4 ± 4.4	95.2 ± 1.4
C3	0.42 ± 0.00	12.31 ± 0.75	1073 ± 20	2.05 ± 0.00	No residual strain	87.9 ± 7.0	94.9 ± 0.4
C4	0.55 ± 0.00	10.00 ± 0.00	801 ± 10	2.56 ± 0.00	No residual strain	78.1 ± 1.1	97.8 ± 0.1
C5	1.12 ± 0.00	4.98 ± 0.00	380 ± 10	2.56 ± 0.00	No residual strain	27.6 ± 1.9	66.0 ± 1.2
C6	0.29 ± 0.00	24.97 ± 7.67	1710 ± 224	3.41 ± 0.02	No residual strain	89.5 ± 1.3	97.5 ± 1.5
C7	0.35 ± 0.00	26.01 ± 1.16	1770 ± 270	3.69 ± 0.04	No residual strain	98.0 ± 4.8	97.9 ± 1.8
C8	0.10 ± 00	N/A	N/A	N/A	No residual strain	94.9 ± 4.1	99.0 ± 3.5
C9	0.28 ± 0.00	15.81 ± 0.35	1530 ± 60	2.88 ± 0.00	No residual strain	88.1 ± 2.1	90.9 ± 2.9
C10	0.26 ± 0.0	22.90 ± 3.10	1910 ± 490	2.07 ± 0.0	Residual strain	79.4 ± 20.0	88.8 ± 19.5
C11	0.31 ± 0.00	24.63 ± 3.39	1715 ± 100	3.85 ± 0.02	No residual strain	98.1 ±1.3	96.5 ± 1.9
C12	0.29 ± 0.00	30.23 ± 0.63	2120 ± 120	3.87 ± 0.02	No residual strain	85.0 ± 16.0	97.0 ± 6.2

S3. Performance comparison to state-of-the self-healing materials

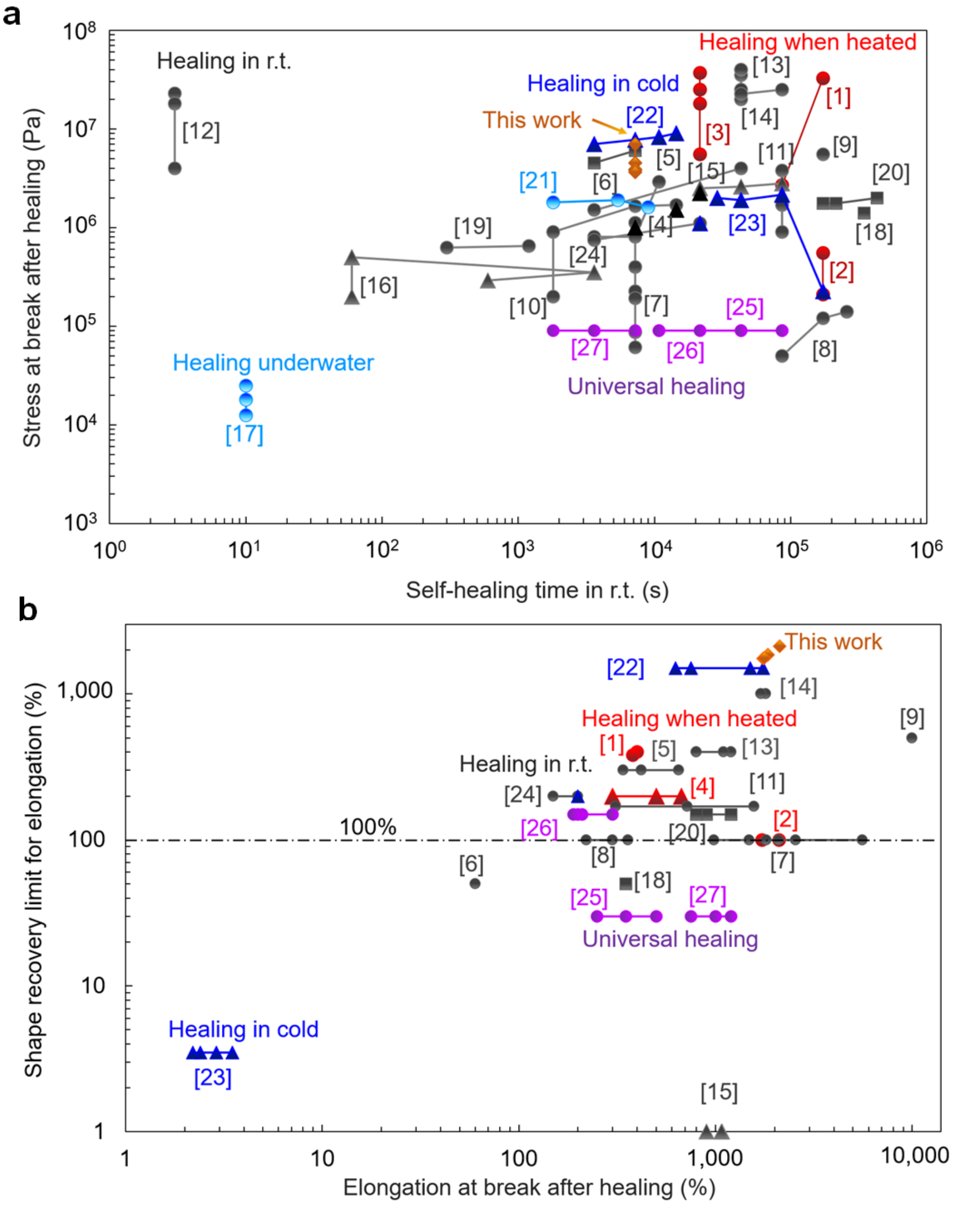

Figure S7. Performance comparisons for state-of-the-art self-healing materials. (a-b) Hydrogen bonds, metal-ligand interactions, and other (such as ionic and combined effects) are denoted by circles, triangles, and squares, respectively. Colour coding for self-healing materials are as follows: elevated temperature (red), room temperature (less than 30 °C) (grey), cold conditions (below 0 °C) (dark blue), underwater (light blue), and universal conditions (purple). Underwater self-healing materials that do or do not heal in saline water are classified as the same. Materials that were classified as a universal healing material could heal in at least four different dry and wet conditions in addition to room temperature (less than 30 °C). Self-healing times are given in seconds at room temperature (which may vary between 20-30 °C). Shape recovery is given as maximum elongation that materials could be elongated when either fully recovering their original shape, or to point where negligible residual strain existed (less than ~20%). References for the figures are given in **Table S3**.

Table S3. List of references for Figure S7.

Data	Reference
**1**	Eom, Y., Kim, SM., Lee, M. et al. Mechano-responsive hydrogen-bonding array of thermoplastic polyurethane elastomer captures both strength and self-healing. Nat Commun 12, 621 (2021). https://doi.org/10.1038/s41467-021-20931-z
**2**	Zhang, Z., Ghezawi, N., Li, G., Ge, S., Zhao, S., Saito, T., Hun, T., Hun, D., Cao, P.-F., Autonomous self-healing elastomers with unprecedented adhesion force. Adv. Funct. Mater., vol. 31, iss. 4, 2170025 (2021). https://doi.org/10.1002/adfm.202170025
**3**	Yang, S., Wang, S., Du, X., Du, Z., Cheng, X., Wang, H., Mechanically robust self-healing and recyclable flame-retarded polyurethane elastomer based on thermoreversible crosslinking network and multiple hydrogen bonds. Chemical Engineering Journal, vol. 391, 123544 (2020). https://doi.org/10.1016/j.cej.2019.123544
**4**	Cao, L., Gong, Z., Liu, C., Fan, J., Chen, Y., Design and fabrication of mechanically strong and self-healing rubbers via metal-ligand coordination bonds as dynamic crosslinks. Composites Science and Technology, vol. 27, 108750 (2021). https://doi.org/10.1016/j.compscitech.2021.108750
**5**	Cordier, P., Tournilhac, F., Soulié-Ziakovic, C. et al. Self-healing and thermoreversible rubber from supramolecular assembly. Nature 451, 977–980 (2008). https://doi.org/10.1038/nature06669
**6**	Wu, J., Cai, L.-H., Weitz, D. A., Tough self-healing elastomers by molecular enforce integration of covalent and reversible networks. Adv. Mater., vol. 29, iss. 38, 1702616 (2017). doi: 10.1002/adma.201702616
**7**	Cao, P.-F., et al., Superstretchable, self-healing polymeric elastomers with tunable properties. Adv. Funct. Mater., vol. 28, iss. 22, 1800741, 2018. doi: 10.1002/adfm.201800741
**8**	Tang., M., Zheng, P., Wang, K., Qin, Y., Jiang, Y., Cheng, Y., Li, Z., Wu, L., Autonomous self-healing, self-adhesive, highly conductive composites based on a silver-filled-polyboronsiloxane/polydimethylsiloxane double-network elastomer. J. Mater. Chem. A, 7, 27278-27288 (2019). https://doi.org/1meta0.1039/C9TA09158K
**9**	Lei, Z., Wu, P. A highly transparent and ultra-stretchable conductor with stable conductivity during large deformation. Nat Commun 10, 3429 (2019). https://doi.org/10.1038/s41467-019-11364-w
**10**	Wu, T., Chen, B., Synthesis of multiwalled carbon nanotube-reinforced polyboronsiloxane nanocomposites with mechanically adaptive and self-healing capabilities for flexible conductors. ACS Appl. Mater. Interfaces, 8, 36, 24071-24078 (2016). https://doi.org/10.1021/acsami.6b06137
**11**	Chen, Y., Kushner, A., Williams, G. et al. Multiphase design of autonomic self-healing thermoplastic elastomers. Nature Chem 4, 467–472 (2012). https://doi.org/10.1038/nchem.1314
**12**	Pena-Francesch, A., Jung, H., Demirel, M.C. et al. Biosynthetic self-healing materials for soft machines. Nat. Mater. 19, 1230–1235 (2020). https://doi.org/10.1038/s41563-020-0736-2
**13**	Wang, Y., Huang, X. & Zhang, X. Ultrarobust, tough and highly stretchable self-healing materials based on cartilage-inspired noncovalent assembly nanostructure. Nat Commun 12, 1291 (2021). https://doi.org/10.1038/s41467-021-21577-7
**14**	10. Li, Y., Li, W., Sun., A., Jing, M., Liu, X., Wei, L., Wu, K., Fu, Q., A self-reinforcing and self-healing elastomer with high strenght, unprecedented toughness and room-temperature reperability. Mater. Horiz., 8, 267 (2021). doi: 10.1039/d0mh01447h
**15**	Lai, JC., Jia, XY., Wang, DP. et al. Thermodynamically stable whilst kinetically labile coordination bonds lead to strong and tough self-healing polymers. Nat Commun 10, 1164 (2019). https://doi.org/10.1038/s41467-019-09130-z
**16**	Wang, C., Liu, N., Allen, R., Tok, J. B.-H., Wu, Y., Zhang, F., Chen, Y., Bao, Z., A rapid and efficient self-healing thermo-reversible elastomer crosslinked with graphene oxide. Adv. Mater., vol. 25, iss. 40, 5785-5790 (2013). https://doi.org/10.002/adma.201302962
**17**	7. Chen, W.-P., Hao, D.-Z., Hao, W.-J., Guo, X.-L., Jiang, L., Hydrogel with ultrasfast self-healing property both in air and underwater. ACS Appl. Mater. Interfaces, 10, p. 1258-1265 (2018). doi: 10.1021/acsami.7b17118
**18**	Lou, Z., Liu, Z., Guo, Y., Lei, D., You, Z., A new strategy of discretionarily reconfigurable actuators based on self-healing elastomers for diverse soft robots. Adv. Funct. Mater., vol. 31, iss. 11, 20082328 (2021). https://doi.org/10.1002/adfm.202008328
**19**	Xu. C, Cao, L., Lin, B., Liang, X., Chen, Y., Design of self-healing supramolecular rubbers by introducing ionic cross-links into natural rubber via a controlled vulcanazation. ACS Appl. Mater. Interfaces, 8, 27, 17728-17737 (2016). https://doi.org/10.1021/acsami.6b05941
**20**	Niu, W., Cao, X., Wang, Y., Yao, B., Zhao, Y., Chen, J., Wu, S., Zhang, S., He, X., Photonic vitrimeter elastomer with self-healing, high toughness, mechanochromism, and excellent durability based on dynamic covalent bond. Adv. Funct. Mater., vol. 31, iss. 13, 2009017 (2021). https://doi.org/10.1002/adfm.202009017
**21**	Davydovich, D., Urban, M.W. Water accelerated self-healing of hydrophobic copolymers. Nat Commun 11, 5743 (2020). https://doi.org/10.1038/s41467-020-19405-5
**22**	Li, CH., Wang, C., Keplinger, C. et al. A highly stretchable autonomous self-healing elastomer. Nature Chem 8, 618–624 (2016). https://doi.org/10.1038/nchem.2492
**23**	Lai, JC., Li, L., Wang, DP. et al. A rigid and healable polymer cross-linked by weak but abundant Zn(II)-carboxylate interactions. Nat Commun 9, 2725 (2018). https://doi.org/10.1038/s41467-018-05285-3
**24**	Liu, Z., et. al., Biomimetic materials with multiple protective functionalities. Adv. Funct. Mater., vol. 29, iss. 28, 1901058 (2019). doi: 10.1002/adfm.201901058
**25**	Cao, Y., Wu, H., Allec, S.I., Wong, B.M., Nguyen, D.-S., Wang, C., A highly stretchy, transparent elastomer with the capability to automatically self-heal underwater. Adv. Mater., vol. 30, iss. 49 (2018). doi: 10.1002/adma.201804602
**26**	Cao, Y., Tan, Y.J., Li, S. et al. Self-healing electronic skins for aquatic environments. Nat Electron 2, 75–82 (2019). https://doi.org/10.1038/s41928-019-0206-5
**27**	Guo, H., Han, Y., Zhao, W. et al. Universally autonomous self-healing elastomer with high stretchability. *Nat Commun* **11,** 2037 (2020). https://doi.org/10.1038/s41467-020-15949-8
**28**	Kim, S.-M., Jeon, H., Shin, S.-H., Park, S.-A., Jegal, J., Hwang, S.Y., Oh, D.X., Park, J., Superior toughness of fast self-healing at room temperature engineered by transparent eleastomer. Adv. Mater., vol. 30, iss. 1, 1705145 (2018). https://doi.org/10.1002/adma.201705145

Table S4. A colour-coded comparison table for state-of-the-art self-healing elastomers. The lighter colour means the better the criteria are met. The darkest colour indicates the criteria are not met at all. (N/A = not applicable or not mentioned). References for the table are given in **Table S5**.

					Healing conditions						
**Interaction**	**Mechanically robust**	**Transparent**	**Shape recovery**		**r.t.**	**Water**	**Saline water**	**Cold**	**Supercooled water**	**pH 1-14**	**Ref**
Hydrogen bond			For ~1,800% strain								This work
Hydrogen bond											1
Hydrogen bond											2
Hydrogen bond											3
Hydrogen bond											4
Hydrogen bond											5
Hydrogen bond											6
Hydrogen bond			N/A								7
Hydrogen bond			N/A								8
Hydrogen bond											9
Hydrogen bond			N/A								10
Hydrogen bond											11
Hydrogen bond											12
Hydrogen bond											13
Metal-ligand			For 1,500% strain								14
Metal-ligand											15
Metal-ligand			For 3.5% strain								16
Metal-ligand			For 200% strain								17
Multiple bonds											18

Table S5. List of references for Table S4.

Data	Reference
**1**	Cordier, P., Tournilhac, F., Soulié-Ziakovic, C. et al. Self-healing and thermoreversible rubber from supramolecular assembly. Nature 451, 977–980 (2008). https://doi.org/10.1038/nature06669
**2**	Cao, Y., Wu, H., Allec, S.I., Wong, B.M., Nguyen, D.-S., Wang, C., A highly stretchy, transparent elastomer with the capability to automatically self-heal underwater. Adv. Mater., vol. 30, iss. 49 (2018). doi: 10.1002/adma.201804602
**3**	Wu, J., Cai, L.-H., Weitz, D. A., Tough self-healing elastomers by molecular enforce integration of covalent and reversible networks. Adv. Mater., vol. 29, iss. 38, 1702616 (2017). doi: 10.1002/adma.201702616
**4**	Cao, P.-F., et al., Superstretchable, self-healing polymeric elastomers with tunable properties. Adv. Funct. Mater., vol. 28, iss. 22, 1800741, 2018. doi: 10.1002/adfm.201800741
**5**	Lei, Z., Wu, P. A highly transparent and ultra-stretchable conductor with stable conductivity during large deformation. Nat Commun 10, 3429 (2019). https://doi.org/10.1038/s41467-019-11364-w
**6**	Chen, Y., Kushner, A., Williams, G. et al. Multiphase design of autonomic self-healing thermoplastic elastomers. Nature Chem 4, 467–472 (2012). https://doi.org/10.1038/nchem.1314
**7**	Chen, W.-P., Hao, D.-Z., Hao, W.-J., Guo, X.-L., Jiang, L., Hydrogel with ultrasfast self-healing property both in air and underwater. ACS Appl. Mater. Interfaces, 10, p. 1258-1265 (2018). doi: 10.1021/acsami.7b17118
**8**	Pena-Francesch, A., Jung, H., Demirel, M.C. et al. Biosynthetic self-healing materials for soft machines. Nat. Mater. 19, 1230–1235 (2020). https://doi.org/10.1038/s41563-020-0736-2
**9**	Eom, Y., Kim, SM., Lee, M. et al. Mechano-responsive hydrogen-bonding array of thermoplastic polyurethane elastomer captures both strength and self-healing. Nat Commun 12, 621 (2021). https://doi.org/10.1038/s41467-021-20931-z
**10**	Li, Y., Li, W., Sun., A., Jing, M., Liu, X., Wei, L., Wu, K., Fu, Q., A self-reinforcing and self-healing elastomer with high strenght, unprecedented toughness and room-temperature reperability. Mater. Horiz., 8, 267 (2021). doi: 10.1039/d0mh01447h
**11**	Zhang., Z., Ghezawi, N., Liu, B., Ge, S., Zhao, S., Saito, T., Hun, D., Cao, P.-F., Autonomous self-healing elastomers with unprecedented adhesion force. Adv. Funct. Mater., vol. 31, iss. 4, 2006298 (2021). doi: 10.1002/adfm.202006298
**12**	Cao, Y., Tan, Y.J., Li, S. et al. Self-healing electronic skins for aquatic environments. Nat Electron 2, 75–82 (2019). https://doi.org/10.1038/s41928-019-0206-5
**13**	Wang, Y., Huang, X. & Zhang, X. Ultrarobust, tough and highly stretchable self-healing materials based on cartilage-inspired noncovalent assembly nanostructure. Nat Commun 12, 1291 (2021). https://doi.org/10.1038/s41467-021-21577-7
**14**	Li, CH., Wang, C., Keplinger, C. et al. A highly stretchable autonomous self-healing elastomer. Nature Chem 8, 618–624 (2016). https://doi.org/10.1038/nchem.2492<br>15. Lai, JC., Jia, XY., Wang, DP. *et al.* Thermodynamically stable whilst kinetically labile coordination bonds lead to strong and tough self-healing polymers. *Nat Commun* **10,** 1164 (2019). https://doi.org/10.1038/s41467-019-09130-z
**15**	Lai, JC., Jia, XY., Wang, DP. *et al.* Thermodynamically stable whilst kinetically labile coordination bonds lead to strong and tough self-healing polymers. *Nat Commun* **10,** 1164 (2019). https://doi.org/10.1038/s41467-019-09130-z
**16**	Lai, JC., Li, L., Wang, DP. et al. A rigid and healable polymer cross-linked by weak but abundant Zn(II)-carboxylate interactions. Nat Commun 9, 2725 (2018). https://doi.org/10.1038/s41467-018-05285-3
**17**	Liu, Z., et. al., Biomimetic materials with multiple protective functionalities. Adv. Funct. Mater., vol. 29, iss. 28, 1901058 (2019). doi: 10.1002/adfm.201901058
**18**	Guo, H., Han, Y., Zhao, W. et al. Universally autonomous self-healing elastomer with high stretchability. Nat Commun 11, 2037 (2020). https://doi.org/10.1038/s41467-020-15949-8

S4. Stress relaxation and mechanical hysteresis in elastomers

The relaxation times were measured at room temperature with rate of 25%s^{-1} (**Figure S8**). The relaxation times are indicated as time required for the stress to decrease to 1/e. The relaxation times were significantly increased as the length of the long polymer chains increased and when bimodal chain length distribution varied (Figure S8a-b). The relaxation times were found to be dependent on the elongation due to bimodality of the network and reinforcing effect of nanoparticles (varied between 25-218 seconds for 100-500% strains) (Figure S8b-c).

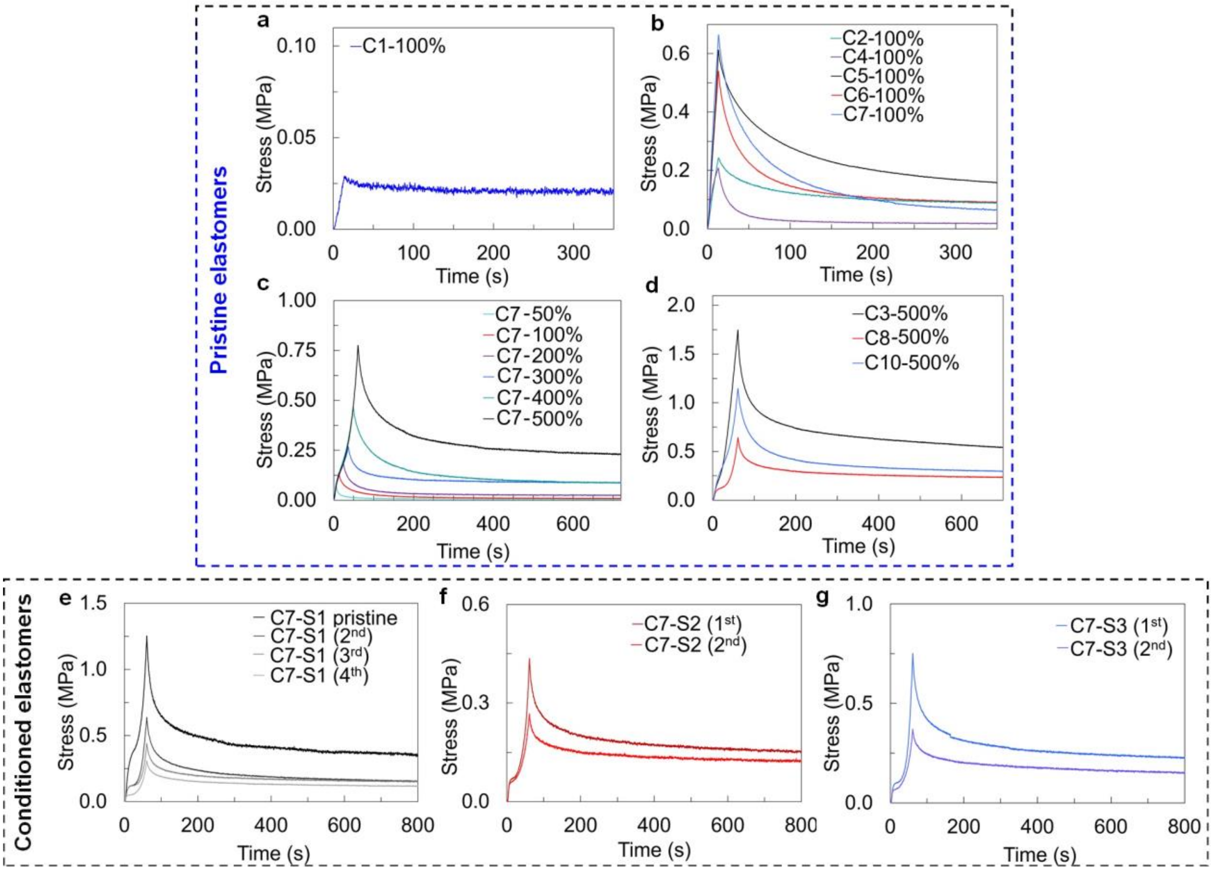

Figure S8. a-d) Stress-relaxation for pristine elastomers at 50-500% strain. e-g) Stress-relaxations for pristine and conditioned elastomer when elongated to 500% strain (where S1 denotes specimen 1). e) S1 was conditioned by elongation to 500% and holding the static load for at least 500 seconds, f) S2 was elongated once to its breaking point, and g) S3 was cycled at 500% strain for at least 10 times. e-g) The specimens were healed for 2 hours at room temperature between stress relaxation tests.

The mechanical stress-strain hysteresis was measured at room temperature with rate of 25 $\%s^{-1}$ (**Figure S9**). The magnitude of mechanical hysteresis was found to relate to end-to-end distance of long polymer chains, bimodal chain length distributions, and degree of chemisorption between nanoparticles and polymer (which is affect by curing temperature). By changing the bimodal chain length distribution (number of short chains increased), mechanical hysteresis significantly increased (Figure S9a-c). With lower curing temperature and/or increased amount of cross-linking component (hard phase), a reverse effect was observed (Figure S9d-e). By increasing the amount of boron oxide nanoparticles similar mechanical hysteresis existed (Figure S9e and S9g). The mechanical hysteresis significantly increases at large strains as there can also exist friction with the disentanglement of interpenetrated network when chain segments orientate parallel to stress. However, local molecular rearrangements (due to supramolecular interactions) are mainly responsible for the

mechanical hysteresis, especially in low strain regions (because disentanglements are known to take orders of magnitude longer).

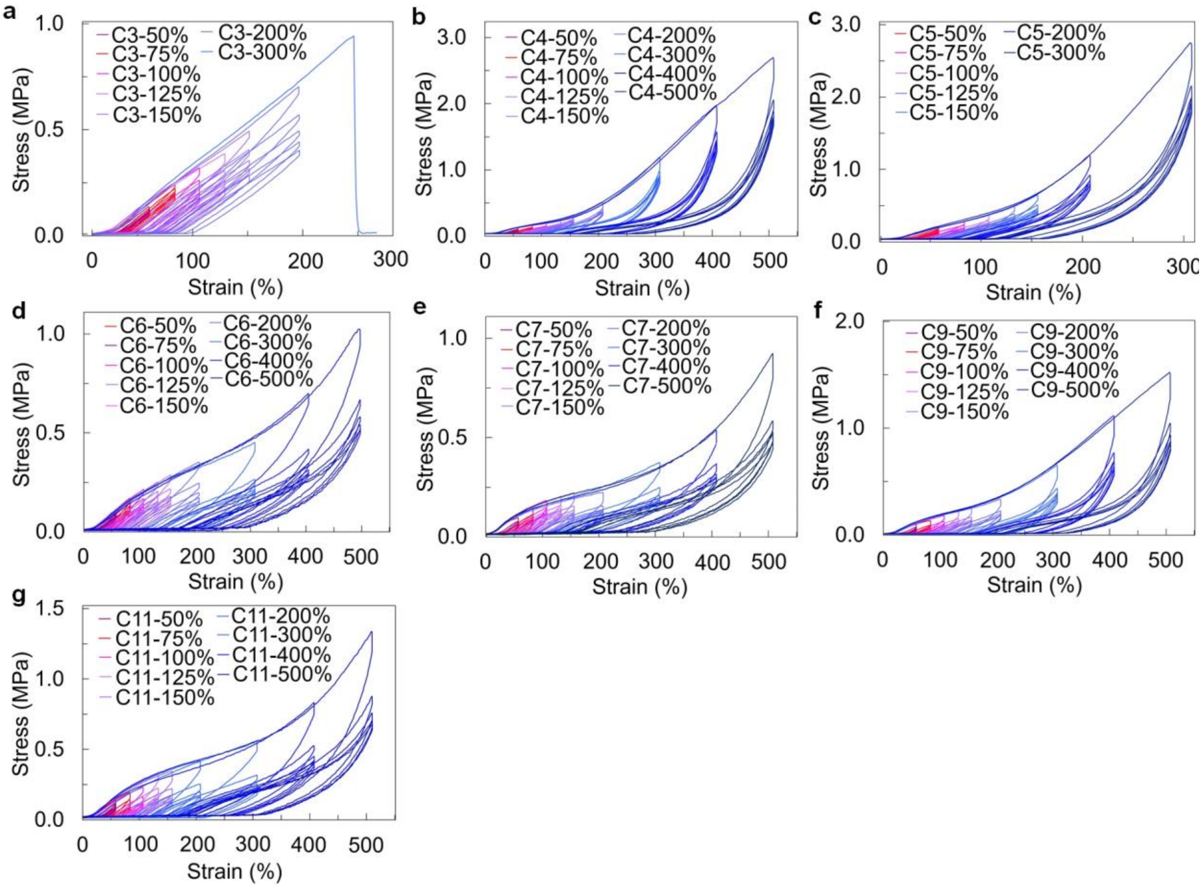

Figure S9. Stress-strain hysteresis was measured with pristine elastomers for 5 consecutive cycles at 50-500% strain. Following compositions were measured: a), C3, b) C4, c) C5, d) C6, e) C7, f) C9, and g) C11.

The relaxation times were measured at room temperature with rate of 25%s^{-1} (**Figure S8**). The relaxation times are indicated as time required for the stress to decrease to 1/e. The relaxation times were significantly increased as the length of the long polymer chains increased and when bimodal chain length distribution varied (Figure S8a-b). The relaxation times were found to be dependent on the elongation due to bimodality of the network and reinforcing effect of nanoparticles (varied between 25-218 seconds for 100-500% strains) (Figure S8b-c).

S5. Mechanical conditioning effects in elastomer.

Changes in relaxation times, mechanical hysteresis and strain-induced reinforcements were evident with specimens that were repeatedly elongated over the plastic region of hard phase (even as healing times significantly increased) (**Figure S10**). Thus, it was found that specific

elastomer compositions could be mechanically conditioned (denoted as C7, C11, C12) (**Table S1**) as their *E*, σ_{break}, and ε_{break} values became relatively constant after repeatedly broken. Such conditioning process has been undiscovered in any other self-healing materials up to date. We hypothesize this could relate to existing microphase-separated morphology in the elastomers with a particular composition (e.g., a specific composition of hard phase and a low cross-linking temperature).

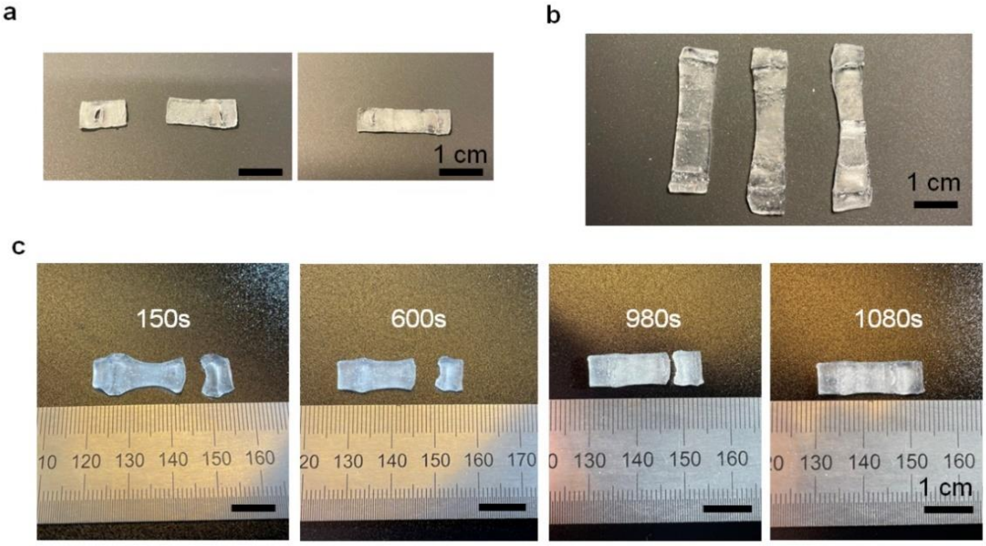

Figure S10. a-c) Photographs of specimen fractured into two pieces after elongation to their breaking point at rate of 5%s^{-1}. a) Two notches existed due to large compressive stress from the static clamps. The notches gradually decreased in a size, and afterwards fractured surfaces were aligned for healing. b) Three specimens were elongated to break and healed at room temperature after manually put together. The specimens were elongated to break approximately 10 minutes apart (leftmost healed for ~20 minutes in photographs). c) A specimen was elongated to break and left to recover its shape. After most of the shape has recovered, the fractured surfaces fit well together.

The relaxation times (τ_r) for the best overall performing composition (C7) increases from 180 to 740 seconds as a specimen was conditioned with static load (**Figure S8e**). When dynamically conditioned, the relaxation times increased to over 740 seconds with smaller number of conditioning cycles (Figure S8f-g). Because the τ_r of the bimodal elastomers increased through conditioning, they can become significantly more structurally stable (in comparison to pristine elastomers with short relaxation times). Thus, elastomers can be adapted to withstand extremely large deformations at rapid rate ($\varepsilon(t)$) because the τ_r of the conditioned bimodal network did not exceed the critical relaxation (τ_{cr}) time even with high strain rates. Accordingly, as critical relaxation time of the network is exceeded ($\tau_r > \tau_{cr}$) (dependent on the structure of elastomer), the network would fracture.

It was found that a specimen could lose significantly more toughness (in comparison to the best-case scenario) when excessively elongated over their ε_{break}. Static and dynamic condition were significant slower if the tensile stress during condition was less than 50% of ε_{break} (**Figure S11**). However, a slower conditioning process may not lead to a different result in terms of mechanical properties in the final specimen. During the conditioning process there was no difference (in terms of elastomers mechanical properties) whether elongated at maximum rate of the tester (25%s^{-1}) to breaking point or at slower rate (such as 5%s^{-1}). In both cases, the elastomers were stabilized after two elongation-healing cycles leading to similar result in terms of its mechanical properties (E, σ_{break} and ε_{break}).

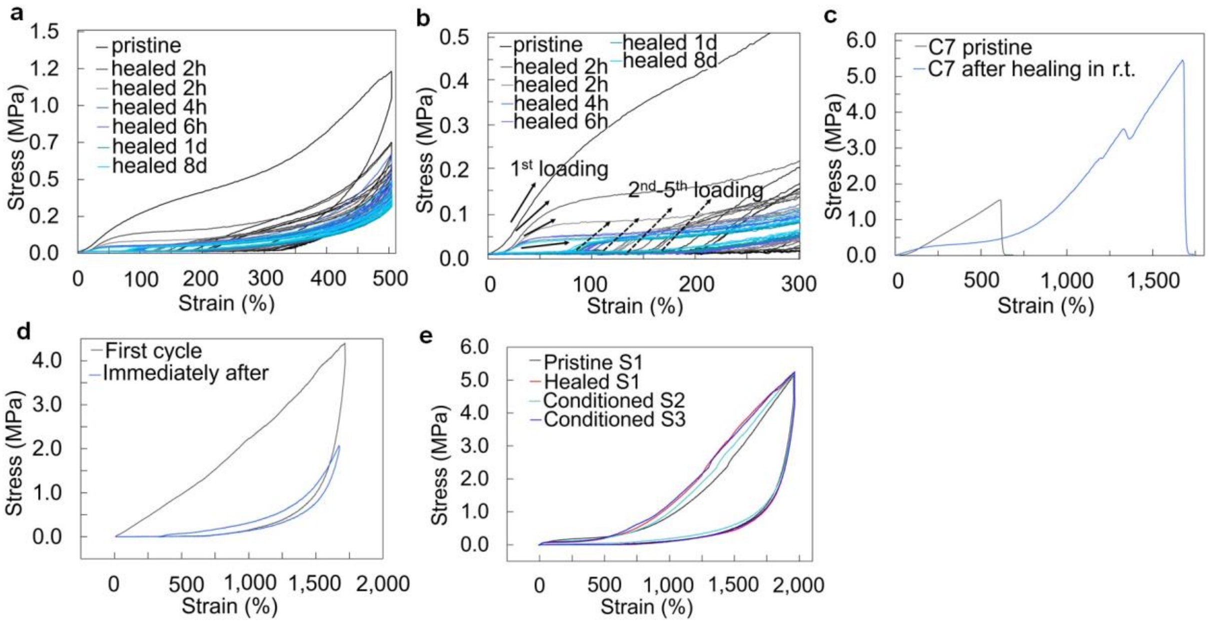

Figure S11. a-b) Mechanical hysteresis measured by elongating to 500% strain for 5 cycles within each measurement times and healed with varied times in-between measurements. c) Pristine C7 elongated to break two times (at rate 25%s^{-1}). d) Pristine C7 measured for two consecutive stretching-releasing cycles. e) Mechanical hysteresis measured (at rate 25%s^{-1}) for pristine and conditioned specimens (by different methods). S1 was elongated to ε_{break} before the second measurement. S2 was cycled at 500% strain for 10 cycles before measurement. S3 was conditioned with static load for 3 times (at 500% strain) for 800 seconds each time before measurement. It was found that toughness could be increased up to ~30 MJm^{-3} as curing time increases from 12 to 24 hours in C7.

It is assumed that the conditioning process leads to "reconfiguration" of the bimodal interpenetrated network because dilatant characteristics of pristine elastomers are lost, and they adapt to withstand large deformations at rapid rates. The reconfiguration of the conditioned bimodal network would increase the time required for the chain segments to relax which then leads to extremely long relaxation times under large stress (in comparison to other flexible polymers). It is further assumed that there are permanent changes in the viscoelastic

components (Helmholtz free energy and rate of energy dissipation) because shape recovery times also changed due to mechanically conditioning. It is likely that breakage of non-ideal permanent junction points occurs in the system while the material is being mechanically conditioned. In this case, “non-ideal” means permanent junction points (or covalent bonds) located in unfavorable positions for deformation to take place. As evidence, sudden changes in stress could appear while the material was elongated (Figure S11c) suggesting breakage of covalent bonds of the hard phase (responsible for the strength of the network in low strain region). These ‘non-ideal’ permanent junctions’ points are expected to be located along contour length of the short chains. Due to entropy-driven elasticity, it is expected that nonaffine deformation exist due to bimodality in the network leading to first deformation of the short chains at low strains. One factor that could be associated with strain-induced reinforcement becoming more visible with conditioned specimens can be partly due to variable Poisson’s ratios leading to discrepancies in true stresses. As the stress was calculated based on assumption that Poisson’s ratio remained constant. Also, conditioning process may change the bimodal chain length distribution which could increase the strain-induced reinforcement of the network. Further aspects of the conditioning process will be studied in detail in the future

S6. Self-healing mechanism in dry and wet conditions

A self-healing mechanism was observed by optical microscopy during the healing process in air and under water (**Video S5 and S6**). It was found that underwater self-healing mechanism could be inherently different depending on whether the cut-surfaces were aligned or left unaligned. For instance, a self-healing rate is dependent how a specimen is damaged in the first place as the elastomer tends to minimize free energy by swelling or deswelling (similar to that in resilin). When elastomers are at their equilibrium (**Figure S12**), uniaxial elongation causes them to swell more while compressing causes them to swell less, respectively.

The elastomer is not fully hydrophobic, but rather has a complex combination of more hydrophilic domains and more hydrophobic domain’s due to the bimodality of the interpenetrated three-dimensional network consisting of two type of dynamic bonds. These combinations can slow the rate of self-healing (**Figures S13 and S14**) when certain conditions were met, for example when cut-surfaces are fully separated. At the start of water immersion, water is energetically favorable for the system until entropic forces are overcome by the elastic forces of the elongated chains.

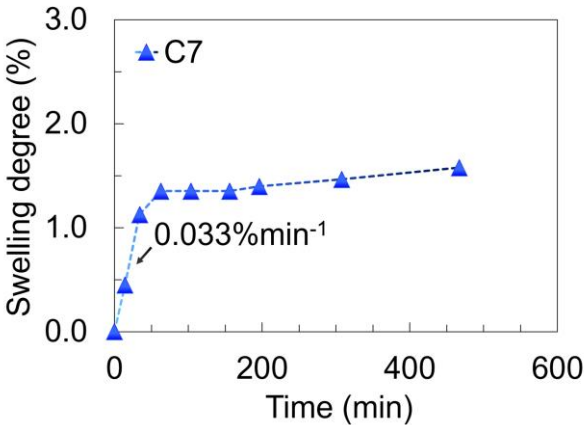

Figure S12. Swelling degree of the C7 elastomer underwater (water temperature ~23 °C, ambient humidity ~50.35%). Factors such as ambient humidity may have an effect to the swelling degree of the elastomers as their equilibrium changes. It should be pointed that the weight the elastomers did not change with change in ambient humidity (< 55%).

During uptake of water molecules, the equilibrium of the condensation/hydrolysis reaction changes and the dynamic interaction of the supramolecular Si-O:B dative bonding increases. It is assumed that Si-O:B dative bonding is responsible for the reversible transition between the softened-hardened states, and efficient self-healing in presence of ambient humidity, wet conditions and underwater. The condensation/hydrolysis equilibrium improves dynamic interaction of Si-O:B dative bonding as additional free functional groups can appear. However, this may not be enough to overcome the hindered physical contact and saturation of hydrogen bonds in cut-surfaces in presence of water molecules (due to hydrophobic domains interaction) in shorter time scales. In prolonged water immersion, an external force may be required to enable physical contact and fast wetting because elastomers have tendency to float in an aqueous medium. With proper physical contact, the self-healing may proceed as in other occasions following the main five stages of crack healing.

Hydrophobic domains and improved dynamic interactions of O-B bonds can facilitate improved self-healing rate when healing without intervention (Figure S14). One of the major reasons is the temporary softening of elastomer which leads to more formation of tacky surfaces (that is a result of the improved dynamics of O-B bonds). As the elastomer tries to minimize its free energy by retracting (when manually bisected), this facilitates even faster self-healing because physical contact of cut-surfaces is maintained (not manually separated). Elastomers at swelling equilibrium are increasingly more prone to larger deformation with similar magnitude of force (Equations S6-S7). This increases self-healing rate with prolonged underwater immersion due to elastomer tendency to minimize free energy and achieve

equilibrium by any means. Also, any degree of swelling can be beneficial to reduce the physical distance between damaged locations and enable more efficient wetting. On short time scales hydrophobic domains may even slow down the absorption of water molecules (beneficial for wetting and diffusion of dynamic bonds).

It should be pointed out that the underwater healing without intervention mechanism is feasible in applications where materials are unable to come into physical contact for wetting (no actuation). With some appropriate form of mechanical actuation, both mechanisms can be meaningful dependent on the conditions in which self-healing occurs.

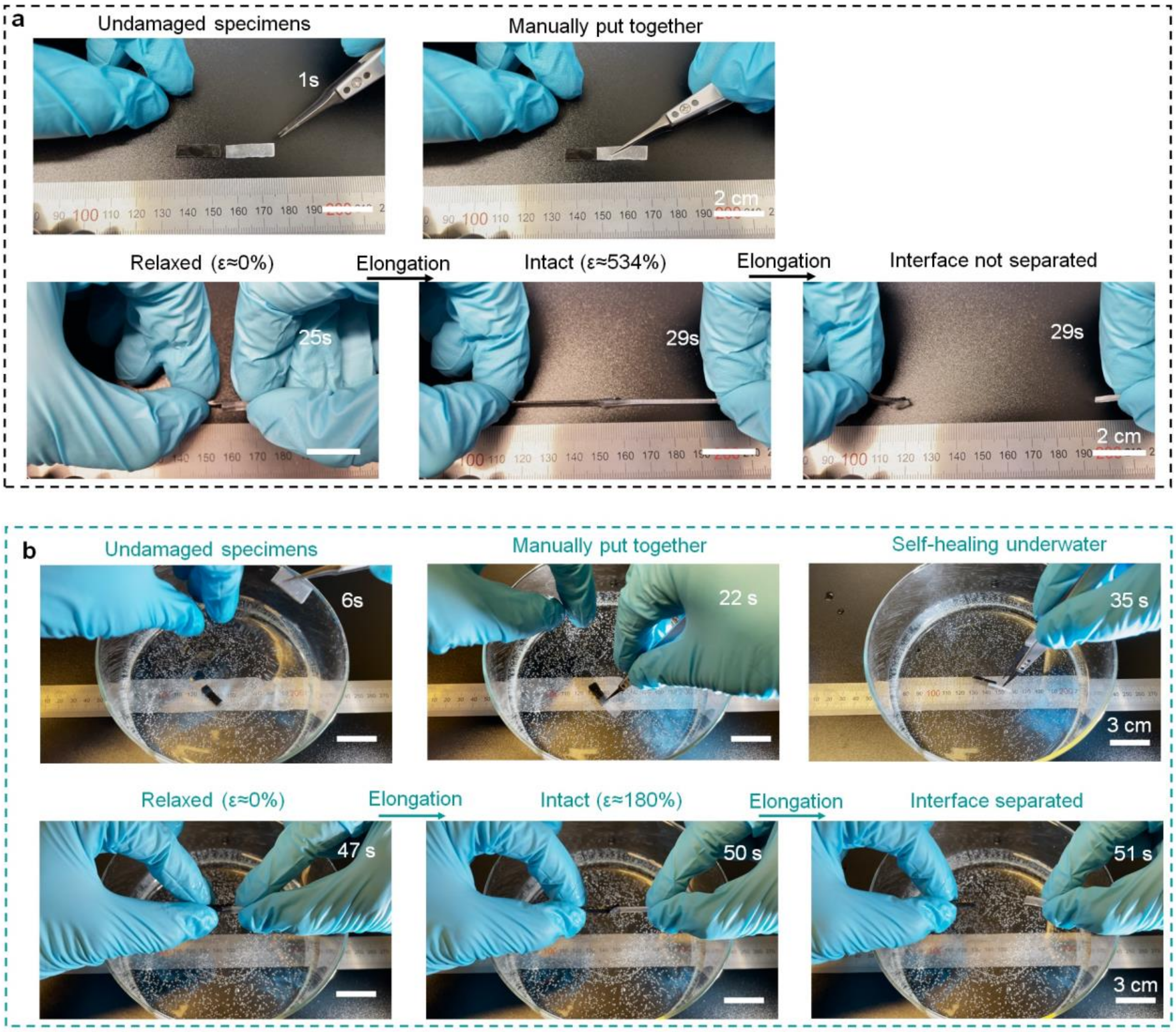

Figure S13. a-b) Photographs showing as undamaged specimens were self-bonded together in air and under water (20 °C). The interface healed in remained intact when elongated to 534% strain at rate of 120 $\%s^{-1}$. The underwater healed interface broke when strain reached 180% at rate of $60\%s^{-1}$. The specimens were stacked from their ends in both cases for self-bonding.

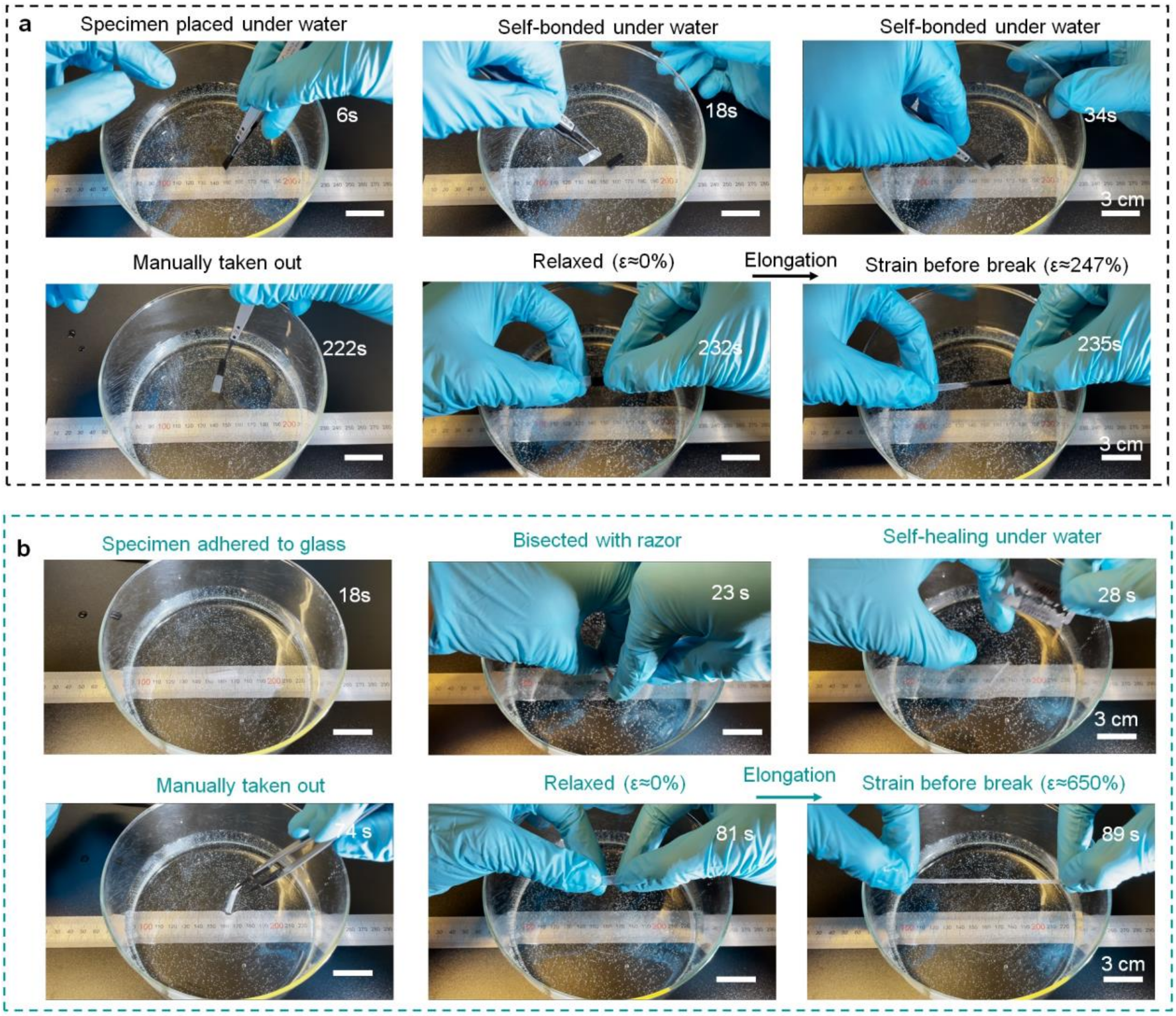

Figure S14. a) Photographs showing underwater healing for undamaged specimens placed under water for less than 5 minutes. After healing for ~200 seconds under water, specimens could be elongated to ~150-230% strain at rate ~50-80%s^{-1}. Test was repeated multiple times with similar results when surfaces were aligned withing 30 seconds. As self-healing times decreased (to ~30 seconds) or time taken before alignment increased (>30 seconds), the specimens could withstand only elongation to 40-100% strain (at rate varying from ~50-80%s^{-1}). b) Photographs showing healing for a pristine specimen placed under water and then bisected with razor (but not manually separated or aligned). The specimen was then kept under water for ~50 seconds before elongated to break. The healed elastomer withstood elongation to ~500% at rate of ~60%s^{-1}.

S7. Discussion and advantages of mechanochromic sensor

Self-healing mechanochromic materials are an intriguing research direction in the future as their responses can be recorded in real-time (including modern smart phones) bypassing any design complexities. Electrical transducing modes (such as piezoresistivity) require variety of additional electronic components (data acquisition, signal processing/transmission, displays, etc.), and may or may not even require continuous external energy inputs to be able to collect, transmit, and display information.

Existing material technologies have inherent limitations for building robust self-healing multicomponent systems. As self-healing is added value it should not compromise the performance of a component or material. The performance needs to match or exceed of its non-healable counterpart (please see Table S4). Majority of state-of-the-art self-healing materials are not able to match their properties compared to non-healable counterparts (in aspects of sensitivity, stability, response times, etc.)[1]. This is due to insufficient mechanical performance of the self-healing materials (lack resilience, high mechanical hysteresis, and so forth) (please see Figure S7b). While poor electrical performance (poor sensitivity, non-linearity, etc.) often relates to the typical composite-approach (planar structures) and not taking advantage of microstructural designs (as in non-healable sensors)[2].

It can also be a particularly challenging task to simultaneously achieve stability and self-healing in harsh environmental conditions with multicomponent systems. Even though it has been shown that partial or full recovery of a structural integrity and functionality of individual component is possible, this does not yet ensure self-healability of a multicomponent system. Individual components may have complex surface chemistries (dependent on the nature of their dynamic bonds) and their mechanical compliances may vary. Then, restoring structural integrity of a system by self-healing becomes a difficult challenge as delamination and compliance mismatches pose significant risks for non-recoverable failures (beyond what is possible to be self-healed). Similar issues with mechanochromic materials may not exist due to simplified designs (as in this case).

Another challenge in soft sensors relying on electrical transducing modes is incorporation of multimodal features and improve spatial resolution to measure localized events and magnitude of stimuli accurately[1]. It is believed more focus should be devoted to exploring self-healing mechanochromic sensors as most of the design complexities of multicomponent systems can be bypassed. Mechanochromic materials (especially cholesteric mesophases) are already responsive to various stimuli and may be already capable of providing a higher spatial resolution than their electrical counterparts (as is the case here). For

instance, electronic transducing modes require a passive or active sensing matrix to incorporate a spatial resolution whose density is limited by numerous factors (such as magnitude of parasitic elements, increased transient response times) due to the electronic components. Such limiting factors do not exist in mechanochromic materials where a large continuous surface is already responsive to numerous localized events with high resolution[3]. Aspects of multimodality in mechanochromic sensors may be solvable with appropriate sensor design and further tuning of the mesophase composition in the future (such as incorporation of dual functionality with electrical filler).

Non-healable mechanochromic sensors often have insufficient fatigue resistance. This will lead to loss of functionality in longer time periods. It is possible to increase fatigue resistance by incorporation of mechanochromic functionality into the self-healing material[4] (as shown here). Due to the large viscosity of the hydrated cholesteric mesophase it does not leak out from the sensor when a sensor is punctured, torn, or bisected in various harsh environmental conditions. It is assumed a similar design approach is directly applicable to liquid metal based self-healing devices. However, the transition from the hydrated to the dry cholesteric mesophase is a major challenge (in terms of stability) due to water permeability (an issue that exists both in non-healable and healable sensors). This can lead to loss of coloration on longer time scales[5] which can be significantly faster with materials having a certain type of reversible dynamic chemistry. Herein, it was found that the cholesteric mesophase transitioned into the dry state within less than 10 days (when the surface area of the chromic layer was less than 3 cm^2).

The operation range of a non-healable sensor relying on electrical or optical transducing modes (especially those relying on microstructural design of nanomaterials) is often severely limited to elongations less than 100%. It was shown that the operating range of the mechanochromic sensor was extended up to 300% strain. The most limiting factor with any mechanochromic materials can be their slow response times. It was found that visual coloration fully recovered in roughly 0.5 to 40 seconds after removal of stress (which is dependent on multiple factors). For instance, recovery of coloration can be significantly slower after a large compressive stress than with uniaxial elongation. This relates to the nature of dynamic bonds that slows down the shape recovery process (as layers compress against each other). Full recovery of visual colorations could be faster than 0.5s when sensors were adhered to the supporting structure (such as human skin) and used as wearable sensors. Further improvements in the response times would be possible by decreasing the thickness and/or modulus of the sensor which simultaneously could improve sensitivity to low strains.

In such attachments, the improvement in response times could be accounted for by the fact that a self-adhered sensor follows deformation of the supporting structure that it is adhered to. This aids in stress relaxation and shape recovery in the low strain region[4].

It is expected that issues with moisture sensitivity, poor responsiveness to small forces (in comparison to non-healable counterparts with electrical transducing modes), slow response times (applicability only to low frequency motion less than 10 Hz), and stimuli-decoupling can be addressed in future work to widen applicability of the materials for wearable sensing.

References for this section:

1. Khatib, M., Zohar, O., and Haick, H. Self-healing soft sensors: from materials design to implementation. Adv. Mater., 33, 2004190 (2021). https://doi.org/10.1002/adma.202004190
2. Ruth, S.R.A., Feig, V.R., Tran, H., and Bao, Z. Microengineering pressure sensor active layers for improved performance. Adv. Funct. Mater., 30, 2003491 (2020). https://doi.org/10.1002/adfm.202003491
3. Liang, HL., Bay, M.M., Vadrucci, R. et al. Roll-to-roll fabrication of touch-responsive cellulose photonic laminates. Nat Commun 9, 4632 (2018). https://doi.org/10.1038/s41467-018-07048-6
4. Wang, Y., Shang, L., Chen, G., Sun, L., Zhang, X., and Zhao, Y. Bioinspired structural color patch with anisotropic surface adhesion. Sci. Adv., 6, eaax8258 (2020). https://doi.org/10.1126/sciadv.aax8258
5. Kamita, G., Frka-Petesic, B., Allard, A., Dargaud, M., King, K., Dumanli, A.G., and Vignolini, S. Biocompatible and sustainable optical strain sensors for large-area applications. Adv. Optical Mater., 2016, 4, pp. 1950-1954. https://doi.org/10.1002/adom.201600451

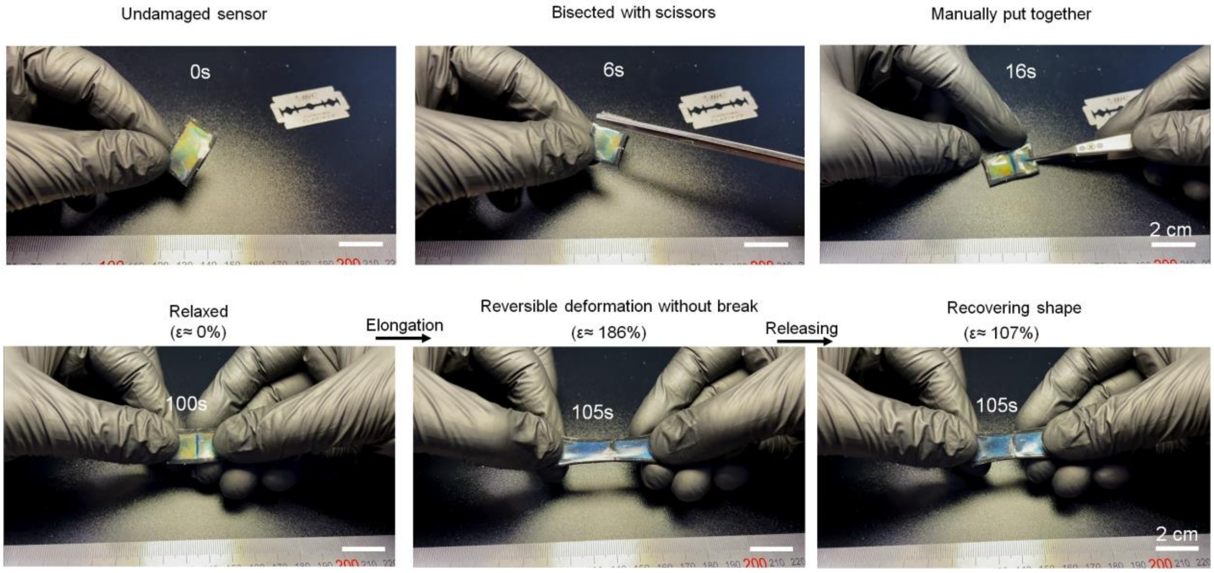

Figure S15. Self-healing at room temperature when bisected with scissors and manually put together following damage. The mechanochromic sensor was healed for ~80 seconds and afterwards it could withstand elongation to ~186% strain without breaking (at rate ~40%s^{-1}). The specimen recovered its shape after the stress was released. The cut location is visible as

dark blue indicating that the interface is not fully healed in such a short period of time (as expected).

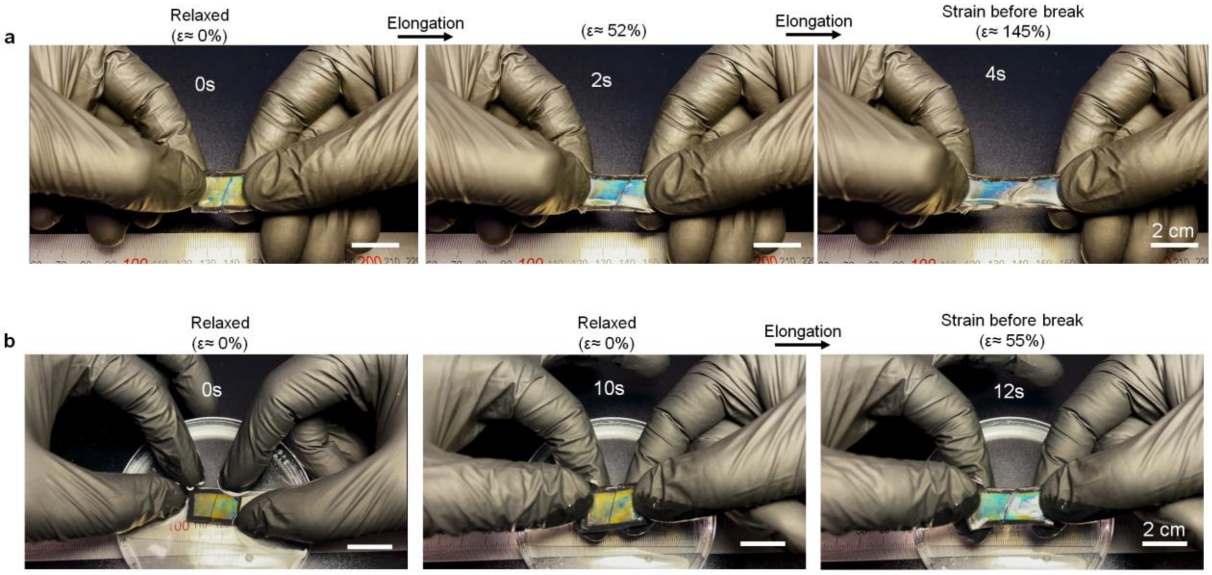

Figure S16. a-b) Self-healing at room temperature and under water without intervention (not manually put together following a damage). After ~300 seconds of healing, the mechanochromic sensors could withstand elongation of ~55-145% without breaking (at rate ~25-40%s^{-1}). The self-healing rate is very different on small time scales (as in other cases) in comparison to when manually put together (due to lack of force input or external energy).
